# Stable Neutralization of a Virulence Factor in Bacteria Using Temperate Phage in the Mammalian Gut

**DOI:** 10.1128/mSystems.00013-20

**Published:** 2020-01-28

**Authors:** Bryan B. Hsu, Jeffrey C. Way, Pamela A. Silver

**Affiliations:** a Department of Systems Biology, Harvard Medical School, Boston, Massachusetts, USA; b Wyss Institute for Biologically Inspired Engineering, Boston, Massachusetts, USA; c Department of Biological Sciences, Virginia Tech, Blacksburg, Virginia, USA; University of California San Diego

**Keywords:** Shiga toxin, bacteriophage, microbiome, antivirulence

## Abstract

Elimination or alteration of select members of the gut microbiota is key to therapeutic efficacy. However, the complexity of these microbial inhabitants makes it challenging to precisely target bacteria. Here, we deliver exogenous genes to specific bacteria by genomic integration of temperate phage for long-lasting modification. As a real-world therapeutic test, we engineered λ phage to transcriptionally repress Shiga toxin by using genetic hybrids between λ and other lambdoid phages to overcome resistance encoded by the virulence-expressing prophage. We show that a single dose of engineered phage propagates throughout the bacterial community and reduces Shiga toxin production in an enteric mouse model of infection without markedly affecting bacterial concentrations. Our work reveals a new framework for transferring functions to bacteria within their native environment.

**IMPORTANCE** With the increasing frequency of antibiotic resistance, it is critical to explore new therapeutic strategies for treating bacterial infections. Here, we use a temperate phage, i.e., one that integrates itself into the bacterial genome, to neutralize the expression of a virulence factor by modifying bacterial function at the genetic level. We show that Shiga toxin production can be significantly reduced *in vitro* and in the mammalian gut. Alternative to traditional applications of phage therapy that rely on killing bacteria, our genetics-based antivirulence approach introduces a new framework for treating bacterial infections.

## Abstract (precis)

With the increasing frequency of antibiotic resistance, it is critical to explore new therapeutic strategies for treating bacterial infections. Here, we use a temperate phage, i.e., one that integrates itself into the bacterial genome, to neutralize the expression of a virulence factor by modifying bacterial function at the genetic level. We show that Shiga toxin production can be significantly reduced *in vitro* and in the mammalian gut. Alternative to traditional applications of phage therapy that rely on killing bacteria, our genetics-based antivirulence approach introduces a new framework for treating bacterial infections.

## INTRODUCTION

The human gut microbiota is a collection of microbes colonizing the gastrointestinal tract and has been associated with various aspects of human health (1). While this community typically works in concert with our bodies, substantial perturbations such as antibiotics or infections can disrupt the microbial balance and lead to long-lasting dysbiosis (2). In some instances, pathogenic bacteria do so by transmitting virulence factors to commensal bacteria through plasmid-based (3) and phage-based (4) horizontal gene transfer (HGT). Remediating diseases associated with these pathogens while minimizing unintended and disruptive effects on the surrounding microbiota remains challenging (5), especially with the limited tools available for targeting particular species (6).

Our ability to manipulate the composition and function of the gut microbiota is presently limited in terms of precision and durability (6). Antibiotics nonspecifically decimate swaths of gut species (7), dietary changes affect both the overall microbiota and the mammalian host, probiotics poorly engraft due to colonization resistance (8), and even highly specific lytic phages can cause unintended changes in the bacterial community despite targeting specific species (9). While in some cases these strategies may show transient efficacy, the emergence of resistant mutants can impact therapeutic effect. Broadly resetting the gut microbiota through fecal microbiota transplants (FMTs) has been promising especially for treating Clostridium difficile infections (10), but they are difficult to characterize and may transmit unintended traits such as obesity (11).

An alternative strategy is to modify bacterial function within its native environment. For example, one approach has been to develop drugs that target the virulence factors of antibiotic-resistant pathogens to specifically neutralize their deleterious effects while minimizing selection for resistance. Although a number of antivirulence drugs are under investigation, the targets for inhibition are generally limited to those accessible by small molecules and biologics (i.e., surface-bound and secreted proteins), may require multiple drugs targeting multiple virulence factors, and could have off-target effects on other microbes and the host (12). While the principle of antivirulence is attractive, it remains challenging in application.

Shiga toxin (Stx)-producing Escherichia coli is one example of a pathogenic infection that is challenging to treat. Antivirulence drugs targeting the toxin have been investigated but failed clinical trials (13). Antibiotics are contraindicated because of their potential to exacerbate virulence (14). While there are multiple virulence factors in the foodborne pathogen enterohemorrhagic E. coli (EHEC), Stx is significantly associated with disease severity (15) and can lead to hemolytic-uremic syndrome (16). Of the two main Stx variants—Stx1 and Stx2—the latter is ∼1,000-fold more toxic (17). This *stx* gene carried by temperate phages is expressed when the phage undergoes lytic replication. Similarly to a number of other prophage-encoded virulence factors (18), the region in which the *stx* gene is located is not expressed while the phage is in a lysogenic state, i.e., stably integrated into the bacterial genome. It is not until induction, whether occurring spontaneously or from stimuli such as antibiotics, that the lytic life cycle is activated to produce Stx2 (19) and progeny phage that can spread virulence genes to commensal E. coli species (20).

Instead of an antimicrobial strategy for killing pathogens, a genetics-based antivirulence strategy could neutralize virulence before expression and minimize resistance until the bacteria have been completely shed from the gastrointestinal tract. Temperate phages offer a solution as they are genetically engineerable and can integrate into the bacterial chromosome as prophages for long-lasting effects conferring fitness advantages on the bacterial host (21). Instead of relying on a nonnative constituent of the gut that could face practical barriers for efficacy, temperate phages are abundantly found in human gut bacteria (22–24) and can constitute large portions of the bacterial chromosome (25).

Here, we report the use of a genetically engineered temperate phage to repress Stx from an established E. coli population colonizing the mammalian gut. We first show that genetic hybrids between lambdoid phages can overcome phage resistance mechanisms while maintaining function. We then genetically encode a transcriptional repressor of Stx in our engineered phage and show that it substantially reduces Stx produced by E. coli MG1655 *in vitro*. Finally, we demonstrate that our engineered phage, when administered to mice precolonized by this E. coli strain, can propagate throughout the murine gut from a single dose to significantly reduce fecal Stx concentrations. Our work describes a new therapeutic framework for the *in situ* modification of gut bacteria for genetics-based antivirulence.

## RESULTS

### Engineered temperate phage to repress a bacterial virulence factor in the gut.

**FIG 1:**
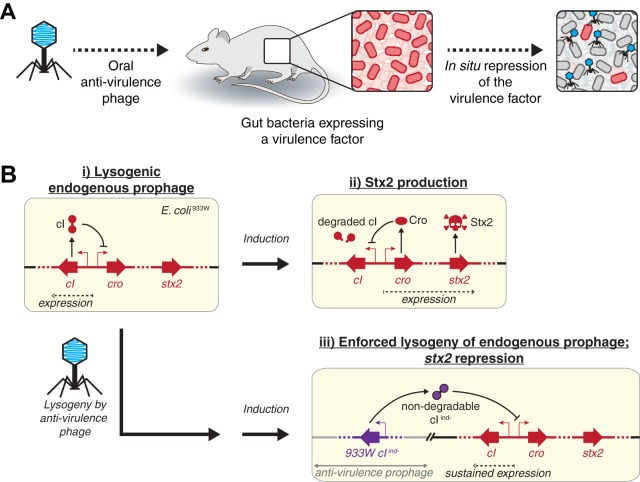
Neutralizing Stx2 production from E. coli using an engineered temperate phage. (A) As an alternative to bacteriolytic strategies that aim to block pathogenesis by killing bacteria, we propose an alternative approach that aims to reduce expression of the virulence factor. We do so by introducing a phage to lysogenize the targeted bacteria within the mammalian gut and to express a transcriptional repressor of the virulence factor. (B) (i) Genetic schemes of E. coli^933W^ showing the 933W prophage expressing *cI* to maintain a lysogenic state in which *stx2* is not expressed and (ii) induction that causes degradation of the cI protein leading to expression of the lytic genes including *cro* and *stx2*. (iii) This leads to cell lysis, releasing phage progeny and Stx2 protein. Expression of a nondegradable *cI* for the 933W prophage, *933W⋅cI^ind-^*, from a genomically integrated engineered temperate phage (antivirulence prophage) can force the 933W prophage to remain lysogenic despite induction and degradation of endogenous cI protein.

Modifying bacterial function with species and genetic-level specificity requires a high level of precision, especially within their natural ecosystem. While the complexity and heterogeneity of the mammalian gut make it an especially challenging environment for targeted modifications, phages have adapted with high bacterial host specificity, making them an attractive therapeutic tool. As shown in Fig. 1A, we used phages not to kill bacteria in the phage therapy approach but to modify a specific function of the targeted bacteria within the mammalian gut. Because antibacterial approaches can enrich for resistance, we aimed to engineer temperate phages to deliver an antivirulence payload that neutralizes virulence without relying on killing the bacteria.

Expression of Stx2 is dependent upon induction of the 933W prophage. To demonstrate the viability of our proposed antivirulence approach, we aimed to neutralize the production of Stx2 from the 933W prophage, which is one of a number of Stx2-producing prophages derived from enterohemorrhagic E. coli strains. As schematically depicted in Fig. 1B, panel i, the 933W prophage in E. coli (E. coli^933W^) maintains a dormant state by expression of its repressor protein, cI, which blocks expression of *cro* and consequently lytic genes including *stx2*. Induction, which occurs spontaneously and by stimuli such as antibiotics, causes activation of the bacterial SOS response and RecA-mediated degradation of cI (26) (Fig. 1B, panel ii). This ultimately leads to expression of the lytic genes that produce phage progeny and Stx2. As the phage-encoded repressor for the 933W prophage, cI, is key to blocking lytic induction and maintaining the dormant lysogenic state (27), we engineered a second exogenous temperate phage to constitutively express a nondegradable mutant of this repressor (*933W⋅cI^ind-^*) that contains a Lys178Asn mutation (27). By maintaining repression despite induction, this neutralizes production of progeny phage and Stx2 (Fig. 1B, panel iii).

**FIG 2:**
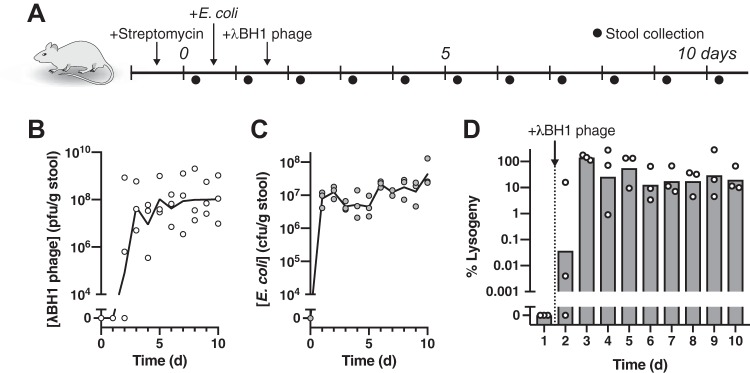
Temperate phage robustly transduces E. coli colonizing the mouse gut. (A) Experimental timeline examining the impact of λBH1 phage on precolonized E. coli in mice. (B) Fecal concentrations of free λBH1 phage and (C) E. coli. (D) Percentage of fecal E. coli lysogenized by λBH1. Symbols represent distinct samples from individual mice (*n *= 3) with lines or bars representing the geometric mean.

To illustrate the feasibility of using a temperate phage, we show that bacteriophage λ transduces a substantial fraction of targeted bacteria in the mammalian gut. As shown in Fig. 2A, we used a streptomycin-treated mouse model to quantitate temperate phage lysogeny on E. coli colonizing the mammalian gastrointestinal tract. One day after colonization with E. coli MG1655, we introduced λBH1 phage by oral gavage and collected daily stool samples for analysis of bacterial and phage titers. We constructed λBH1 from λ phage by inserting an antibiotic resistance cassette for quantification of lysogens. After oral administration of λBH1 phage, we found that fecal phage levels reached equilibrium approximately 2 days later and persisted at substantial concentrations (>10^6^ PFU/g stool) for the duration of the experiment (Fig. 2B). As phage in the absence of its cognate bacterial host is undetectable in the stool of mice ∼2 days after administration (28), our results indicate that λBH1 phage is capable of continuous replication in the gut, enabling its expansion throughout the bacterial population from a single dose. Furthermore, introduction of λBH1 phage did not significantly alter fecal E. coli concentrations (Fig. 2C), which is in sharp contrast to lytic phages that can cause substantial reduction (9). The maintenance of high levels of phage indicates sustainable phage production, likely from a subpopulation of bacteria undergoing spontaneous induction *in vivo*. With one bacterium capable of producing hundreds of wild-type λ phage *in vitro* (29), it is possible that only a fraction of bacteria needs to undergo induction. Using antibiotic selection, we quantified the number of fecal E. coli bacteria harboring the λBH1 prophage and found that a substantial fraction (∼17% to 30%) remained lysogenized by days 7 to 10 (Fig. 2D). Overall, these results indicate that the temperate phage λ is capable of widespread modification of its cognate bacteria in the gut.

### Phage hybridization overcomes superinfection exclusion.

As gut bacteria harbor numerous prophages including those encoding virulence (30), overcoming superinfection exclusion mechanisms is crucial for achieving efficacious *in situ* transduction. In the foodborne pathogen EHEC, the lambdoid prophage 933W both produces Stx2 and inhibits phage superinfection by other lambdoid phages (31).

**FIG 3:**
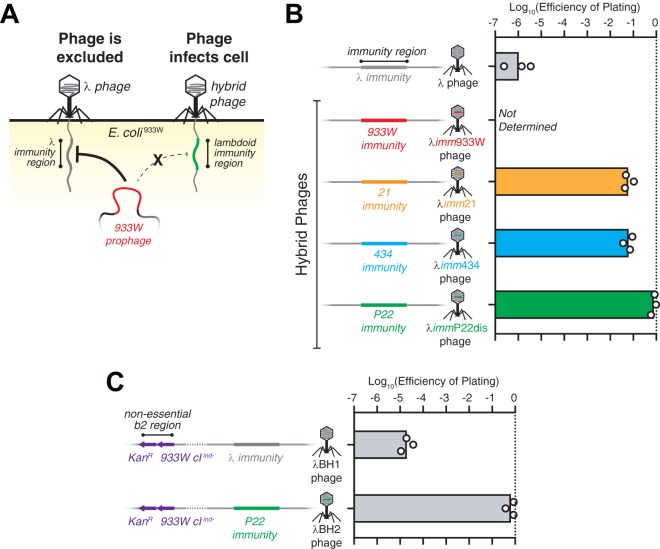
Hybrid λ phages overcome superinfection exclusion by prophage 933W. (A) Depiction of superinfection exclusion by the 933W prophage that inhibits infection by λ phage but is ineffective against a hybrid phage that contains the immunity region from another lambdoid phage in a λ phage background. (B) Schematic representation of a portion of the λ phage genome containing the λ immunity region and its hybrids containing immunity regions from other lambdoid phages (933W, 21, 434, and P22) in a λ phage background. Efficiencies of plating (EOPs) for λ phage and its hybrids on E. coli^933W^ are shown to the right. (C) Schematic representation of λBH1 phage, which is a λ phage with a kanamycin resistance cassette (Kan^r^) and *933W⋅cI^ind-^* inserted into the nonessential *b*2 region of the phage genome. λBH2 phage is a product of a phage cross between λBH1 and λ*imm*P22dis resulting in a phage containing Kan^r^ and *933W⋅cI^ind-^* genes with a P22 immunity region in a λ phage background. Their respective EOPs against E. coli^933W^ are shown to the right. Symbols represent biological replicates with bars representing the geometric mean.

E. coli^933W^ excludes λ phage infection, but genetic hybrids of λ with other lambdoid phages restore infectivity. As shown schematically in Fig. 3A, the 933W prophage inhibits infection from λ phage by recognition of its immunity region, i.e., indispensable genes responsible for the lysis-lysogeny decision in the phage life cycle. Because lambdoid phages have similarities in genetic function and organization despite dissimilar sequences, it is feasible to replace the λ immunity region with orthologous immunity regions from other lambdoid phages to overcome the superinfection exclusion (32). We found that the efficiency of plating (EOP) of λ phage against E. coli^933W^ was ∼10^6^-fold lower than against the nonlysogen (Fig. 3B), confirming its superinfection exclusion. We verified that this effect is not due to a cI-based immunity (see Fig. S1 in the supplemental material). We then tested the EOP for genetic hybrids of λ phage in which the λ immunity region is swapped with that of other lambdoid phages (e.g., 21, 434, and P22) (Fig. S2 and Table S3). As shown in Fig. 3B, these hybrid phages had substantially improved EOPs against E. coli^933W^ with 6.0% for λ*imm*21 and 6.7% for λ*imm*434 phages. Moreover, hybridization with the *Salmonella* phage P22 resulted in near-complete recovery of EOP to 78% for λ*imm*P22dis phage, indicating that lambdoid phages from noncognate bacterial hosts could be a reservoir for genetic orthologs that maintain phage function while circumventing superinfection exclusion mechanisms.

10.1128/mSystems.00013-20.1 FIG S1 Spot testing of λ and λ*imm*933W phage against lawns of their lysogens in E. coli. The zone of lysis (dark circle) on a lawn of E. coli indicates successful phage infection. Both phages are capable of infecting nonlysogens and lysogens of the other phage (i.e., λ phage infecting a λ*imm*933W lysogen and vice versa), indicating that 933W⋅cI and λ⋅cI are not inhibitory to λ phage and 933W phage infection, respectively. Download FIG S1, EPS file, 49.2 MB. Copyright © 2020 Hsu et al. 2020 Hsu et al. This content is distributed under the terms of the Creative Commons Attribution 4.0 International license.

10.1128/mSystems.00013-20.2 FIG S2 Genetic map of immunity regions within λ phage and its hybrids. (A) Genetic map of portions of the λ phage genome with brackets indicating the regions within λ that have been replaced with immunity regions from other lambdoid phages. (B) Within λ*imm*933W phage, the genes homologous to 933W (accession no. NC_000924) are labeled in red and those homologous to λ (NC_001416) are labeled in black. Percent identities for genes less than identical are indicated in parentheses. (C) Within λ*imm*P22dis phage, the genes homologous to P22 (AF217253) are labeled in green and those homologous to λ (NC_001416) are labeled in black. Download FIG S2, EPS file, 3.5 MB. Copyright © 2020 Hsu et al. 2020 Hsu et al. This content is distributed under the terms of the Creative Commons Attribution 4.0 International license.

10.1128/mSystems.00013-20.8 TABLE S3 DNA sequences in this study. Download Table S3, DOCX file, 0.03 MB. Copyright © 2020 Hsu et al. 2020 Hsu et al. This content is distributed under the terms of the Creative Commons Attribution 4.0 International license.

Efficient gene transduction enables the delivery of antivirulence genes. We inserted genes for *933W⋅cI^ind-^* (to repress *stx2* expression) (Fig. 1B) and a kanamycin resistance cassette (to quantitate lysogeny) into the nonessential *b*2 region of λ (26), producing λBH1 (Fig. 3C and Fig. S3). We confirmed that *933W⋅cI^ind-^* expressed from λBH1 was functional (Fig. S4). To overcome superinfection exclusion from the 933W prophage, we utilized a P22 immunity region instead of a λ immunity region. A phage cross between λBH1 and λ*imm*P22dis resulted in the replacement of ∼6 kb of the immunity region of λBH1 with an ∼5-kb portion of that from λ*imm*P22dis while retaining *933W⋅cI^ind-^* and Kan^r^ genes (Fig. 3C, Fig. S3, and Table S3). This new phage, λBH2, showed improved EOP to 90% (Fig. 3C) and demonstrated a functional loss of λ immunity and gain of P22 immunity, as well as expression of functional *933W⋅cI^ind-^* (Fig. S4).

10.1128/mSystems.00013-20.3 FIG S3 Genetic maps of λBH1 and λBH2 phage. Download FIG S3, EPS file, 0.8 MB. Copyright © 2020 Hsu et al. 2020 Hsu et al. This content is distributed under the terms of the Creative Commons Attribution 4.0 International license.

10.1128/mSystems.00013-20.4 FIG S4 Spot assays of 3 μl of ∼10^7^ PFU/ml of λ, λ*imm*933W, λ*imm*434, and λ*imm*P22dis phages against nonlysogenic E. coli or its λ, λBH1, or λBH2 lysogen. Download FIG S4, PDF file, 0.8 MB. Copyright © 2020 Hsu et al. 2020 Hsu et al. This content is distributed under the terms of the Creative Commons Attribution 4.0 International license.

### Antivirulence phage inhibits Stx2 production *in vitro*.

**FIG 4:**
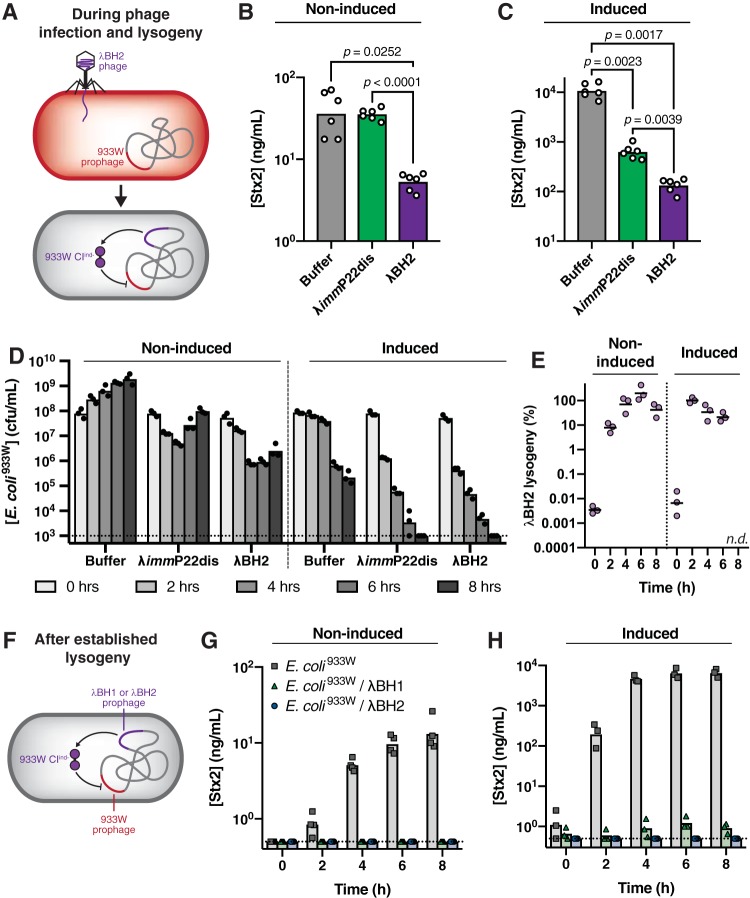
Engineered λ phage neutralizes Shiga toxin production from E. coli^933W^ *in vitro*. (A) E. coli^933W^ was mixed with buffer, λ*imm*P22dis, or λBH2 free phage (MOI of ∼1) at *t* = 0 from which the concentration of Stx2 was measured after 8 h of *in vitro* culture under (B) noninduced and (C) induced conditions (0.5 μg/ml of mitomycin C). Significance was calculated by one-way ANOVA with the *post hoc* Tukey test. (D) Total E. coli^933W^ and (E) the percentage of bacteria lysogenized by λBH2 were measured over 8 h under noninduced and mitomycin C-induced conditions. (F) E. coli^933W^ lysogenized with λBH1 or λBH2 was cultured *in vitro* and analyzed for Stx2 produced under (G) noninduced or (H) induced conditions. Symbols represent biological replicates with bars or lines representing the geometric mean.

Transcriptional repression delivered by λBH2 phage neutralizes Stx2 production. As outlined in Fig. 4A, we tested the efficacy of λBH2 phage to inhibit Stx2 production from E. coli^933W^ by mixing them at equal concentrations (multiplicity of infection [MOI] of ∼1) and culturing for 8 h. We found significantly less Stx2 produced in E. coli^933W^ cultures treated with λBH2 phage than in those untreated (“buffer”) or treated with λ*imm*P22dis phage, the parental phage of λBH2 that is capable of infecting E. coli^933W^ but lacks the *933W⋅cI^ind-^* gene (Fig. 4B). Quantification of bacterial concentrations over time shows that E. coli^933W^ steadily grows over 8 h in the absence of phage (“buffer”) whereas introduction of λ*imm*P22dis results in an initial drop in titer during the first 4 h followed by a recovery (Fig. 4D, noninduced). For λBH2 phage, a similar drop in bacterial concentration was associated with increased lysogenic conversion of E. coli^933W^ that reached 70% by 4 h, indicating that both decreased bacterial titers and repressed *Stx2* expression may contribute to the overall reduction of Stx2 concentration. To confirm that the latter provides sustained antivirulence effect, we isolated λBH2 lysogens of E. coli^933W^, i.e., E. coli containing prophages of both 933W and λBH2 (Fig. 4F) and measured the Stx2 produced in culture. While an E. coli^933W^ culture accumulated 13.1 ng/ml of Stx2 over 8 h, no Stx2 was detected from λBH2 lysogens (Fig. 4G). Similarly, λBH1 lysogens did not produce detectable concentrations of Stx2 despite their poor ability to initially infect E. coli^933W^, confirming that once lysogenic conversion occurs, the resultant lysogens do not produce Stx2.

Stx2 repression is maintained under inducing conditions. DNA-damaging agents such as antibiotics can induce lambdoid prophages toward lysis by activating the bacterial SOS response, leading to RecA-mediated degradation of cI (27). To test whether λBH2 phage remains effective under these more aggressive lytic conditions, we measured Stx2 produced in cultures of λBH2 phage mixed with E. coli^933W^ (Fig. 4A) in the presence of an inducing agent, mitomycin C. As shown in Fig. 4C, E. coli^933W^ receiving buffer alone produces substantially more Stx2 when incubated with mitomycin C due to induction of the 933W prophage. This induction also directs other phages toward primarily lytic replication, and so the introduction of λ*imm*P22dis phage significantly reduces E. coli^933W^ concentrations (Fig. 4D, induced) and consequently Stx2 concentrations (Fig. 4C). Ultimately, λBH2 phage treatment achieves significantly lower Stx2 concentrations than those measured for buffer and λ*imm*P22dis phage conditions (Fig. 4C) because it is capable of repressing *stx*_2_ expression from a large fraction of E. coli^933W^ as shown by the substantial lysogenic conversion (Fig. 4E, induced). Whether an established single lysogen or double lysogen, mitomycin C induction led to lysis after 3 to 4 h (Fig. S5). To confirm that, once lysogeny is established by λBH1 or λBH2 phage, *stx*_2_ repression is maintained even during induction, we cultured λBH2 lysogens of E. coli^933W^ for up to 8 h in the absence or presence of mitomycin C (Fig. 4F). In the case of λBH2 lysogens of E. coli^933W^, we were unable to detect the toxin, indicating that repression is maintained under both noninducing (Fig. 4G) and inducing (Fig. 4H) conditions.

10.1128/mSystems.00013-20.5 FIG S5 Growth curves of lysogens or nonlysogens grown in the absence or presence of mitomycin C. Download FIG S5, PDF file, 0.04 MB. Copyright © 2020 Hsu et al. 2020 Hsu et al. This content is distributed under the terms of the Creative Commons Attribution 4.0 International license.

### Antivirulence phage reduces Stx2 production *in vivo*.

**FIG 5:**
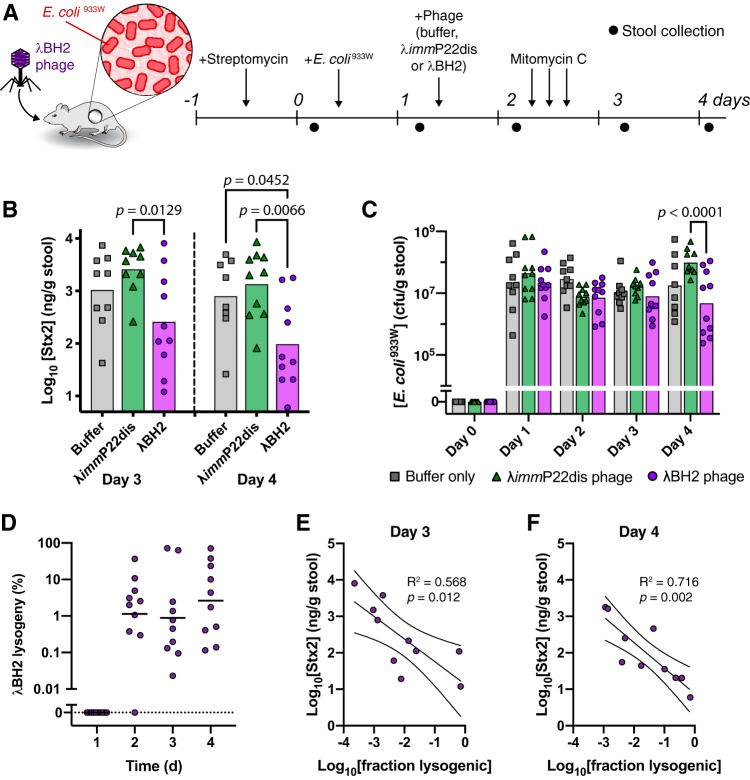
λBH2 phage lysogenizes E. coli^933W^ in the murine gut and reduces fecal Shiga toxin concentrations. (A) Streptomycin-treated mice precolonized with E. coli^933W^ received one dose of 5 × 10^8^ PFU of λ*imm*P22dis phage or λBH2 phage orally. Mitomycin C was administered three times at 3-h intervals by intraperitoneal injection to induce *stx*_2_ expression in the gut. (B) Concentrations of fecal Stx2 after induction with mitomycin C. A one-way ANOVA with *post hoc* Tukey test was used to compare Stx2 levels between buffer, λ*imm*P22dis, and λBH2 conditions while a two-tailed Wilcoxon test was used to compare Stx2 levels after λBH2 phage treatment between days 3 and 4. (C) Concentrations of total fecal E. coli^933W^ with significance calculated by two-way ANOVA with *post hoc* Tukey test and (D) percentage of fecal E. coli^933W^ found to be lysogenized by λBH2 phage. (E) Concentration of fecal Stx2 as a function of fraction of fecal E. coli^933W^ lysogenic for λBH2 phage on day 3 and (F) day 4. Solid and dashed lines represent means and 95% confidence intervals of linear regression, respectively. *P* value describes significance of slope being nonzero. Symbols represent individual mice for buffer (*n* = 9), λ*imm*P22dis (*n* = 10), and λBH2 (*n* = 10) conditions. On days 3 and 4, one λ*imm*P22dis phage-treated mouse and one buffer-treated mouse, respectively, were unable to produce stool for analysis. Bars or lines represent geometric means.

λBH2 reduces fecal Stx2 concentrations in mice. To determine if our phage-based antivirulence strategy is effective *in vivo*, we used a mouse model of enteric Stx2 intoxication from Stx-producing E. coli (33). While it is challenging to model the effect of enteric pathogens, including Stx-producing E. coli, in mice (34), mitomycin C injections can induce substantial quantities of Stx that is otherwise too low to be detected in stool. Mice precolonized by E. coli^933W^ were orally treated with buffer, λ*imm*P22dis phage, or λBH2 phage and then received three doses of mitomycin C by intraperitoneal injection to induce *stx*_2_ expression (Fig. 5A). Daily fecal samples were collected for analysis of bacterial and Stx2 concentrations. After mitomycin C injections, we quantified fecal Stx2 and found that λBH2 phage treatment reduced fecal Stx2 titers compared to buffer and λ*imm*P22dis phage treatment (Fig. 5B). Despite reduced fecal Stx2, all mice began displaying morbidity issues after day 4 and inconsistently produced stool, limiting the duration of the study. Although λBH2 phage did not completely repress Stx2 production, these are highly inducing nonphysiological conditions with fecal Stx2 concentrations (∼10^2^ to 10^3^ ng Stx2/g mouse stool) in excess of those encountered in human Stx-producing E. coli infections (∼2 to 50 ng Stx2/ml human stool) (35).

λBH2 phage lysogenizes E. coli^933W^ and does not affect its titer in the murine gut. Quantification of total fecal E. coli^933W^ generally did not reveal significantly different concentrations between buffer-, λ*imm*P22dis phage-, and λBH2 phage-treated mice (Fig. 5C). Notably, on day 4, mice receiving λ*imm*P22dis phage had significantly higher fecal E. coli^933W^ concentrations than those receiving λBH2 phage and approached significance over buffer treatment (*P = *0.0521). This suggests a fitness benefit from lysogeny with λ*imm*P22dis phage that is not encoded by the λBH2 phage, possibly in a region lost during generation of λBH2 phage by phage cross. Overall, maintenance of high E. coli^933W^ concentrations contrasts with our *in vitro* experiments where induction substantially reduced bacterial concentrations (Fig. 4D) and is likely due to the additional *in vivo* complexities not present in liquid culture such as heterogeneity in mitomycin C exposure and E. coli colonization, dietary and host factors, and influence from the microbiota. Quantification of fecal E. coli^933W^ lysogenized by λBH2 (Fig. 5D) showed that a substantial fraction of the population was transduced, with geometric means between ∼0.9% and 2.6% and individual samples reaching as high as 71%. Notably, there is a large spread in lysogeny between individual mice with daily means lower than what we found in our previous experiment with E. coli lacking the 933W prophage (Fig. 1D). Past work has shown that a high level of induction disfavors lysogeny (36), and within the context of this study, our use of a mitomycin C mouse model may underestimate the achievable degree of lysogeny in more typical, less-inductive conditions such as those shown in Fig. 1.

Lysogeny by λBH2 phage reduces fecal concentrations of Stx2. Though treatment with λBH2 phage shows a reduction in the average fecal Stx2 concentration between treated and untreated groups, we aimed to confirm whether this reduction is indeed associated with lysogenic conversion by λBH2 phage. By plotting the fecal concentration of Stx2 as a function of lysogeny by λBH2, we found that for both days 3 and 4 there is a significantly nonzero inverse correlation (Fig. 5E and F), confirming that reduced fecal Stx2 is caused by the antivirulence effect from λBH2 phage.

## DISCUSSION

Here, we demonstrate a genetic strategy for *in situ* antivirulence treatment of bacteria colonizing the gut. We genetically engineer temperate phage λ to express a repressor that neutralizes Stx production in E. coli and take advantage of the genetic mosaicism of lambdoid phages to create a hybrid phage that is capable of overcoming phage resistance mechanisms. We found that our antivirulence phage not only efficiently infects, lysogenizes, and inhibits Stx2 production from E. coli *in vitro* but is also effective at propagating in the murine gut from a single dose to significantly reduce Stx2 production *in vivo*.

With the complexity and interconnectedness of microbes in the gut, perturbations can have unexpected consequences. Modulating the impact of a bacterial species by manipulating its concentration can lead to unintended effects mediated by interbacterial or bacterium-host interactions. While the typical strategy is to eliminate particular bacteria, it is usually a specific function performed by the bacteria that is deleterious. By precisely and robustly modifying this individual function, a therapeutic effect could persist while minimizing disruption to the surrounding microbiota and avoiding the selection for resistance. Here, we report a framework for making precise genetic modifications that can be practically applied to bacteria within a complex biological system such as the gut microbiome.

For treating pathogenic bacterial infections, disarming their pathogenicity by targeting virulence factors provides a direct therapeutic strategy. Our work represents a step forward; we demonstrate that expression of one such virulence factor, *stx2*, by a laboratory strain of E. coli can be significantly repressed in the murine gut, potentiating its application toward clinically isolated pathogens. With the impending crisis of antimicrobial-resistant infections, new strategies for combating pathogens are desperately needed. Our work illustrates an approach by which bacteria can be specifically modulated *in situ* with rationally designed function in an alternative manner, hopefully inspiring new strategies for treating recalcitrant bacterial infections.

## MATERIALS AND METHODS

### Animal studies.

Animal work was approved by the Harvard Medical School IACUC under protocol number 4966. Female BALB/c mice (Charles River Laboratories) 6 to 7 weeks old were acclimated for 1 week prior to experiments.

To study the effect of temperate phage on nonpathogenic E. coli in the mouse gut (Fig. 2), mice received 5 g/liter of streptomycin sulfate (Gold Bio) in their drinking water, which was replaced every 2 to 3 days. On day 0, 100 μl of streptomycin-resistant E. coli MG1655 was administered to mice by oral gavage. The bacterial gavage solution was prepared from an overnight culture in LB, washed twice with phosphate-buffered saline (PBS), and then diluted 100-fold into PBS, yielding ∼10^7^ CFU/ml. One day later (day 1), mice received 100 μl of λBH1 phage which consisted of a 5 × 10^7^-PFU/ml solution diluted 1:10 into 100 mM sodium bicarbonate immediately prior to gavage. Daily stool samples were collected for microbial quantification. To quantify fecal phage, fresh nonfrozen samples were gently suspended into 1 ml of phage buffer, incubated at 4°C for ∼10 min with a few drops of chloroform, and then pelleted at 4,000 rpm at 4°C. Phage concentration was determined using a double-agar overlay plaque assay (37) in which serially diluted phage solutions were incubated for 20 min at room temperature (RT) with a hardened overlay of E. coli MG1655 in 0.3% agar in tryptone-NaCl-thiamine (TNT) medium over a 1.5% agar in TNT base. After aspiration, plates were incubated at 37°C overnight after which plaques were counted. To quantify fecal E. coli, frozen stool was thawed from −80°C and suspended into 1 ml of PBS by vortexing for 10 min at 4°C followed by low-speed centrifugation at 200 rpm for 20 min to settle fecal debris. The fecal suspension was then serially diluted into PBS, and 100 μl was plated onto MacConkey agar (Remel) plates supplemented with 100 μg/ml streptomycin sulfate to quantify total E. coli or supplemented with 100 μg/ml streptomycin and 50 μg/ml kanamycin to quantify λBH1 lysogens of E. coli.

To study the effect of the engineered temperate phage on Stx2-producing E. coli, mice were treated with similar conditions as described above with the following modifications. On day 0, mice received 100 μl of similarly prepared streptomycin-resistant E. coli^933W^ in PBS by oral gavage. On day 1, mice received 100 μl of λBH2 phage, which was a 5 × 10^10^-PFU/ml solution diluted 1:10 into 100 mM sodium bicarbonate immediately prior to gavage. On day 2, to induce Stx2 expression from engrafted E. coli, mice received three intraperitoneal injections of 0.25 mg/kg of body weight of mitomycin C at 3-h intervals (33). Stool samples were collected daily and stored at −80°C until analysis. Fecal E. coli was quantified by plating as described above, and fecal Stx2 was quantified from the same suspension of stool in PBS by mixing it 10:1 with 20 mg/ml of polymyxin B, incubating it at 37°C for ∼20 min, and then storing it at −20°C until analysis by enzyme-linked immunosorbent assay (ELISA) as described below.

### Bacterial strains.

Bacteria used in this study are listed in Table S1 in the supplemental material. E. coli^933W^ was generated by a previously described method (38), in which 933W phage was produced from the supernatant of a log-phase culture of E. coli O157:H7 strain edl933 in modified LB medium (10 g/liter tryptone, 5 g/liter yeast extract, 5 mM sodium chloride, 10 mM calcium chloride, and 0.001% thiamine) and then stored at 4°C. Molten top agar containing 100 μl of E. coli MG1655 and 3 ml of modified LB medium with 0.3% agar at 45°C was poured onto plates of modified LB agar and allowed to harden. Supernatants of E. coli O157:H7 cultures were then spotted onto the top agar and incubated at 37°C overnight. Resulting plaques were picked and restreaked onto LB. Successful 933W lysogens of E. coli were identified by screens for resistance to λ*imm*933W and the PCR amplification of the *cI*-to-*cro* region of 933W (fwd-AGCCACTCCCTTGCCTCG; rev-GCTTATTTCAAGCATTTCGCTTGC). E. coli lysogens of λ and λ*imm*933W were generated similarly using TNT medium instead of modified LB medium and screened for successful lysogeny by resistance to λ or λ*imm*933W, respectively, and ability to produce phage progeny.

10.1128/mSystems.00013-20.6 TABLE S1 Bacteria used in this study. Download Table S1, DOCX file, 0.01 MB. Copyright © 2020 Hsu et al. 2020 Hsu et al. This content is distributed under the terms of the Creative Commons Attribution 4.0 International license.

### Preparation of high-titer phage stocks.

Phage was propagated via the double agar overlay method where 100 μl of serially diluted phage in phage buffer was mixed with 100 μl of E. coli MG1655 for ∼20 min at RT and then mixed with 3 ml of molten top agar (TNT medium with 0.3% top agar at 45°C) and poured onto prewarmed plates of TNT agar. After incubation overnight at 37°C, top agar from plates with the highest density of plaques was suspended into 5 ml of phage buffer and then gently rocked at 4°C for ∼2 h. Supernatants were sterile filtered to yield ∼10^9^ to 10^10^ PFU/ml of phage. Phage stocks were stored at 4°C.

### Phage strains.

Phage used in this study are listed in Table S2. λBH1 phage was generated using the inherent λred recombination system of λ phage expressed during lytic replication. A crude phage lysate containing recombinant phage was produced by mixing 100 μl of E. coli C600, containing a plasmid vector with Tn*5*-933W⋅cI^ind-^ flanked by 400-bp homology to ea59 and ea47 in a pJET1.2 backbone (Table S3), with 100 μl of serially diluted λ phage; incubated for 20 min at RT followed by addition of 3 ml of molten top agar (TNT medium with 0.3% top agar at 45°C); and poured onto TNT agar plates. After overnight incubation at 37°C, top agar from the plate with the greatest plaque density was resuspended into 5 ml of phage buffer (50 mM Tris, 100 mM sodium chloride, 10 mM magnesium sulfate, and 0.01% gelatin, pH 7.5), sterile filtered, and stored at 4°C. To isolate the recombinant phage, 50 μl of crude phage lysate was mixed with 50 μl of E. coli C600 grown to log phase in LB and incubated for ∼3 h at 37°C. After incubation, 100 μl was plated onto LB containing 50 μg/ml of kanamycin and grown overnight at 37°C with individual colonies restreaked twice. To additionally purify them by plaque purification, colonies were grown overnight in LB and their sterile-filtered supernatants were spotted onto TNT top agar of E. coli C600. Individual plaques were streaked onto LB containing 50 μg/ml of kanamycin and sequenced to confirm insertion in the correct locus of λ phage.

10.1128/mSystems.00013-20.7 TABLE S2 Phages used in this study. Download Table S2, DOCX file, 0.01 MB. Copyright © 2020 Hsu et al. 2020 Hsu et al. This content is distributed under the terms of the Creative Commons Attribution 4.0 International license.

λBH2 phage was generated by a phage cross between λBH1 phage and λ*imm*P22dis phage. Two hundred microliters of a log-phase E. coli C600 culture (7 × 10^7^ CFU/ml) in T broth (1% tryptone and 0.5% sodium chloride) with 0.4% maltose was mixed with a 200-μl solution of λBH1 phage (1.5 × 10^8^ PFU/ml) and λ*imm*P22dis (1.5 × 10^8^ PFU/ml) in phage buffer. After static incubation at 37°C for 20 min, this mixture was diluted 100-fold into prewarmed T broth with 1% glucose and cultured with shaking at 37°C for 90 min. The culture was treated with drops of chloroform and pelleted, and then the supernatant was sterile filtered to produce a crude phage lysate. Residual chloroform was minimized by crossflowing air (Millipore Steriflip) at RT for 1 h. Ten milliliters of this phage lysate was mixed with 1 ml of mid-log culture of a λ lysogen of E. coli C600 grown in LB with 0.4% maltose, incubated at 37°C for 20 min. After ∼30-fold concentration by centrifugation, 200 μl was plated onto LB containing 50 μg/ml of kanamycin. Resulting colonies were restreaked twice and then tested for phage immunity by spot-testing 5 μl of phage against a top agar containing each candidate colony. Correctly engineered phages, as lysogens, were identified from colonies by susceptibility to λ*imm*434 (positive control) but resistance to λ*imm*933W (presence of *933W*·*cI^ind-^* gene) and resistance to λ*imm*P22dis (presence of P22 immunity region). Phage lysates were prepared by culturing colonies overnight in TNT medium, pelleting and sterile filtering the supernatant, and then incubating 100 μl of this phage mixture with 100 μl of E. coli C600 (MOI of ∼0.1) at 37°C for 20 min and then plating it onto LB with 50 μg/ml of kanamycin. After overnight incubation at 37°C, phage was plaque purified by preparing phage lysates from individual colonies as described above and streaking 10 μl onto hardened top agar containing E. coli C600. After overnight incubation at 37°C, individual plaques were picked and restreaked onto LB with kanamycin. The resultant λBH2 lysogen of E. coli was confirmed susceptible to λ and λ*imm*434 as well as resistant to λ*imm*933W and λ*imm*P22dis (see Fig. S4 in the supplemental material). Sequencing confirmed the presence of the P22 immunity region and *933W*·*cI^ind-^* gene (Fig. S3 and Table S3).

### Quantifying phage and efficiency of plating.

The infectivity of phage against E. coli was quantified by the double overlay agar method in which E. coli MG1655 or E. coli^933W^ was cultured overnight in TNT medium, diluted 1:100 into fresh TNT medium, and cultured until mid-log phase of which 50 μl was mixed with 700 μl of molten top agar (TNT medium with 0.5% agar at 45°C) and poured into individual wells of a 6-well plate containing prepoured TNT medium with 1.5% agar. After hardening, 100 μl of phage serially diluted in phage buffer was added and incubated for 20 min at RT followed by aspiration. Plates were incubated at 37°C overnight and then examined for titers of PFU. Efficiency of plating was calculated as the titer of phage on the E. coli^933W^ divided by its titer on the nonlysogenic E. coli.

### *In vitro* assay of phage effect.

E. coli^933W^ was cultured overnight in TNT medium at 37°C, and then cells were washed once with fresh TNT medium and diluted to an optical density at 600 nm (OD_600_) of 0.1 (∼8 × 10^7^ CFU/ml). At *t* = 0, 5 ml of E. coli suspension was mixed with 1 ml of 4 × 10^8^ PFU/ml of λBH2 or λ*imm*P22dis phage solution. To quantify E. coli concentrations in solution, aliquots were collected, serially diluted into PBS, and then spotted (10 μl) onto LB or LB with 50-μg/ml kanamycin plates to quantify total E. coli and λBH2 lysogens, respectively. After 8 h, aliquots were mixed 10:1 with 20 mg/ml of polymyxin B, incubated at 37°C for ∼20 min, and stored at −20°C for quantification of Stx2 by ELISA.

Stx2 concentrations in cultures of E. coli^933W^, its λBH1 lysogen, or its λBH2 lysogen were prepared similarly as described above, where overnight cultures were washed with fresh TNT medium and diluted to an OD_600_ of 0.1. During incubation at 37°C, aliquots were collected, mixed with polymyxin B, and stored at −20°C for quantification of Stx2 by ELISA.

### *In vitro* assay of mitomycin C induction of E. coli lysogens.

Overnight cultures of E. coli (nonlysogen, 933W lysogen, λBH2 lysogen, and 933W/λBH2 double lysogen) in TNT medium at 37°C were diluted to an OD_600_ of 0.1 in fresh TNT medium. The OD_600_ of 100 μl of each sample in a 96-well microtiter plate was then measured with shaking at 37°C continuously for 8 h (BioTek Synergy).

### ELISA quantification of Stx2.

MaxiSorp plates (Thermo Scientific) were incubated with 100 μl/well of mouse monoclonal Stx2 antibody (Santa Cruz Biotechnology; sc-52727) diluted 1:2,500 into PBS for 1.5 h at RT. Plates were washed three times with PBST (PBS with 0.05% Tween 20) and then incubated with 200 μl of 1% bovine serum albumin (BSA) in PBS overnight at 4°C. After washing three times with PBST, 100 μl/well of samples and a standard curve of diluted Stx2 (List Biological Labs; catalog no. 164) were incubated at RT for 2 h. Following sample incubation, plates were washed three times with PBST and 100 μl/well of anti-Stx2 antibody-horseradish peroxidase (Ab-HRP) conjugate was incubated for 1 h at RT. The antibody-enzyme conjugate was previously prepared using an HRP conjugation kit (Abcam; catalog no. ab102890) with a rabbit anti-Stx2 antibody (List Biological Labs; catalog no. 765L) according to the manufacturer’s protocol. After washing three times with PBST, 100 μl/well of colorimetric reagent Ultra TMB (ThermoFisher) was incubated at 37°C for 30 min prior to the addition of 50 μl/well of 2 M H_2_SO_4_ to stop the reaction. Absorbance was measured at 450 nm.

### Data availability.

All materials are readily available from the corresponding authors upon request or are commercially available. There are no restrictions on availability of the material used in the study.
