# Supplementary material for: Stable Neutralization of a Virulence Factor in Bacteria Using Temperate Phage in the Mammalian Gut
Source: mSystems. 2020 Jan 28;5(1):e00013-20. doi: 10.1128/mSystems.00013-20 (PMC6989128; doi:10.1128/mSystems.00013-20)
Supplement: TABLE S3 [file mSystems.00013-20-st003.docx]

| Region | Sequence (5’ to 3’) |
| --- | --- |
| Tn5 and 933WcI^ind-^ genes for insertion into λ. Flanking homology regions for ea59 and ea47 are shown in gray text. The LacUV5 promoter is underlined with mutated nucleotides (uppercase) for constitutive expression. The non-inducible repressor, 933W⋅cI^ind-^ is shown in unformatted black text. A kanamycin resistance cassette (Tn5) is shown in blue text. | agaggttcatctgcatccacctttgctctctgcttttttacgaacattaagcgacttactcgatgcacgcaatggtgtagcaataattgcaactcattccccagtagtactgcaagaggttccaaaatcctgcatgtggaaagtcctacggtcaagagaagcaataaatattatccgtccggatattgagacattcggtgagaacttaggtgttttaactcgtgaggtgtttttacttgaagtgacaaattctggataccaccacttattatcgcagtccgttgattcagagctttcttatgaaaccattctaaaaaattataatggtcagataggattagaaggtcgaaccgttttaaaagcgatgataatgaacagagatgaaggtaaagtacaatgagcgcaacgcaattaatgtgagttagctcactcattaggcaccccaggctttacactttatgcttccggctcgtatAAtgtgtggaattaTgagcggataaTaatttcacacaggaaacagctatggttcagaatgaaaaagtgcgcaaagaattcgcccagcggctagcgcaagcctgtaaagaagctggtcttgatgaacatggtaggggaatggctatagcccgtgccctttctctttcgtccaaaggcgttagcaaatggtttaatgctgagtctttaccgcgtcaggaaaaaatgaatgcgcttgcgaaatttctaaacgttgatgttgtttggcttcagcacggcacttcgttaaatggagcgaatgatgaagatactctttcatttgttggcaaattaaaaaaagggttagtgcgcgtggttggtgaggcaattcttggtgttgatggtgccatcgagatgaccgaagagcgcgatgggtggctcaaaatttatagcgatgatccagatgcctttggtcttcgtgtgaaaggagacagcatgtggcccagaataaaatcaggagaatatgtactcattgagcctaacaccaaagtattcccgggtgatgaggtgtttgtcagaaccgttgaaggacacaacatgattaacgttcttggctatgacagagatggagaataccaatttacaagcattaaccaggatcacaggcctataacgttgccttatcatcaagtagcaaaggtggagtatgtagctggtattctgaagcaatctcgccatctggatgacatcgaggcaagggagtggctgaaaagttcgtgagccctgcaaagtaaactggatggctttcttgccgccaaggatctgatggcgcaggggatcaagatctgatcaagagacaggatgaggatcgtttcgcatgattgaacaagatggattgcacgcaggttctccggccgcttgggtggagaggctattcggctatgactgggcacaacagacaatcggctgctctgatgccgccgtgttccggctgtcagcgcaggggcgcccggttctttttgtcaagaccgacctgtccggtgccctgaatgaactgcaggacgaggcagcgcggctatcgtggctggccacgacgggcgttccttgcgcagctgtgctcgacgttgtcactgaagcgggaagggactggctgctattgggcgaagtgccggggcaggatctcctgtcatctcaccttgctcctgccgagaaagtatccatcatggctgatgcaatgcggcggctgcatacgcttgatccggctacctgcccattcgaccaccaagcgaaacatcgcatcgagcgagcacgtactcggatggaagccggtcttgtcgatcaggatgatctggacgaagagcatcaggggctcgcgccagccgaactgttcgccaggctcaaggcgcgcatgcccgacggcgaggatctcgtcgtgacccatggcgatgcctgcttgccgaatatcatggtggaaaatggccgcttttctggattcatcgactgtggccggctgggtgtggcggaccgctatcaggacatagcgttggctacccgtgatattgctgaagagcttggcggcgaatgggctgaccgcttcctcgtgctttacggtatcgccgctcccgattcgcagcgcatcgccttctatcgccttcttgacgagttcttctaaaattttttatgataaacaattccatccaattgatttaatcaatacaacatttgaagatcaagcagataaatatattttttggcgttatgcagctgacagagccaaaataacaaatgcctatggcttcatttggatatcagagctatggctcagaaaagcaagcatctactccaataaaccaatacatacaatgccaattatagatgaaagacttcaggtaattggaattgattcaaataataatcaaaaatgtatttcatggaaaatagttagagaaaacgaagaaaaaaaaccgactttagaaatatcaacagcagactcaaaacatgacgaaaaaccatatttcatgcgttcagtcttaaaagcaattggcggtgatgtaaacactatgaacaattga |
| Region of 933W phge in λ*imm*933W phage (blue text) flanked by the *cIII* and *ren* of λ (black text). | ttagtctggatagccataagtgtttgatccattctttgggactcctggctgattaagtatgtcgataaggcgtttccatccgtcacgtaatttacgggtgattcgttcaagtaaagattcggaagggcagccagcaacaggccaccctgcaatggcatattgcatggtgtgctccttatttatacataacgaaaaacgcctcgagtgaagcgttattggtatgcggtaaaaccgcactcaggcggccttgatagtcatatcatctgaatcaaatattcctgatgtatcgatatcggtaattcttattccttcgctaccatccattgaaggccatccttcctgaccatttccatcattccagtcgaactcacacacaacaccatatgcatttaagtcgcttgaaattgctataagcagagcatgttgcgccagcatgattaatacagcatttaatacagagccgtgtttattgagtcggtattcagagtctgaccagaaattattaatctggtgaagtttttcctctgtcattacgtcatggtcgatttcaatttctattgatgctttccagtcgtaatcaatgatgtattttttgatgtttgacatctgttcatatcctcacagataaaaaatcgccctcacattggagggcaaagaagatttccaataatcagaacaagtcggctcctgtttagttacgagcgacattgctccgtgtattcactcgttggaatgaatacacagtgcttattcgtactaataaaatacccaattttctgtttcttggttgtgtccaaagttatattcaatatctggtgttgatgtatcaatattcttcataccatcaacaagagttgatacaacagccaaatcttgtttgattctcattaaatggtatttcttccggcgcaataaactttcaatggcaagtttcttcgttgggaatgcaaaagatctttctgcattttttgctactttcttaattgcatatctatttctcctttgtttccattcctgtaaccactgatttggtgctggtttaaaattaacaatccaatgcgcaggaaccaaccatgcataatgctctgtctgatgaaaagctatatattgaagtgcgaatatttttatcccatcttcttcaactgtcgcctggaatctccagaaaacaggcattccatcatgttcagtttctgattcaggaaaaggtacgctccatgattttgtcatatctcacctcaaataagtggtttgctgcctaatttcattttctggcgaccaacacaagtcacctcgccgtcagttgttttgatttccggtagcctgccgcgtaaatggctacgtttggaagacatacaccagtttctggttgcttatgtccaaactcattcgcgtacacaatggccgctcgctccagattgcgtctgtattctttctgttgccagatcacgtcctgtgccatgaacttaattggcttagcgtcttctatgcgctcaggcgtttcgtgagtacctttagcctgaatctgcgctctgcttagagtagggcggtgtaatacttctgaacttattgcttcttcgcgggccagtacgccgttagctaatgcctttgcctttaaacgctcacgacgacgagaacgtgaattgcctttgaactgagttctgcgtgtcatatagacctcctgatgaactttggtggtgtggtaggtgggagacccatttcgacctgtttcggcctacttcaattcggcaatagtcccgcaggcctcgccgctttacgtgcgacatattcccgtccatgaacccttcaccacaccccaaagttcactttggttattgcgctttgtcagcgccgtagattcatattcgaatcgttgtatattcaccgccctggtgagtaatgcgtcctgctgacgacgataataatgaaccaatagttcgacattatcaagaactattggtacgaattttggtgatttattaactctacgaagtatgattctgatatataaggaaatttatttttgaaaatgtggctgatgaaggttatgcggcagggatcataactgcatggtttagcgagttacatcaataaatacaattggttatgttttttaggtgggcgaacgtgaggcaaagaaaacccggcgctgaggccgggctagattttaaagtatttatcttttagagatgtagatgtaaaacttttcgcctttgaaaattttttgtcatcagaagggcttatgaactcatcttttttgtagggaaccgctaatgctgcatcacgtctgcgaggcagcttgcttacttcctcgcgctttttcatgatcagttatcctttaataacctatacagttttgtaggggtacatcctgaggatattgttaagttcgtagcacgccttttccgcccatcatcgtataaacgaaaaccagtagtagacgaattttctgcgtcaaaaactatagacagtatagcgtccccagactttttttgccattcgcatgtgccgttagttggtttcgtcatctgtagacgccagtcaagaacgccatcacttatagctgagagatcgtttagtacatctagtacggattgatatctttcatttggatctacatgaatgcatttgttcactattgttattaattttttatgtatatggggaggatactcttttaatggatagcagccattaattatcgactctctgagttgttcaatcgtgctaaatgcagatctttctctttcaaaattatcatgtccaacacacattctatatatggttaatcctgcctgatatatgtcatatgtgaaattataatcatttgttgataaagaaaaatattccggtggcacatgaaaatgatatccaaactcaggcgcagctctcgattcctcattgactaactgagataatccaaagtcagatagcatggcctcatttctgtttgatatcataatgttattaggttttatatcaaaatgcataagaccttttgagtgtatatgataaagtccacttaaaaattgaatggaataccgtattatctccctgcttgtaagattattttttttcattaattggtttagcgaaccattatgataaaatggcatggctatatagatattgctctcacattgagcagcatactgaacttgcacaatatttggatgtgcatgtttatagagaagccttgcttcattaaagtagtcgtcgtggttagtgttttcttttttttctatttctttaatcaccaagtcatgagctaggtgtctgtcatgagccagatatacttttgaaaaacaaccctgttcttctagatcactaatccattcgaattctacatcagctcttttgtatggagttagcatccccttacctccgcagatagtgcagccaaaacagcttcatttgtttcagttgtaaaaccagaattatcgattccatttatattgcggtgtgacttcaatatttctttatactcgatctctgttaggttcaatgatgatttcatgccagattttctaatagtgtaatattttcttacatcactgcttgaaaatgcttcttgaataacagcttctatataaaggcggtcaatgctaagattatcagagtttgattcagtaacgcgtatggcagctaattcaacattatataaattaagaatgtcgaggatgttatttctcacatactttaatttttctggtgtgtctaaggtcgaaggtattttaataacatcaacacatttgagtgcagactcattagtgcaatatacaacaaaagatgtaactttgggcgccgctctaacacctagtattctcattttttatatcctattttagaatcaggccgcatctctgcgaccatccatcatccaaacgtctcttcactcatccgaagaagcagcaatccgggttagcacgcacaagctcaagcgcatcagtcagcgaaagttcagtactgtactgatgccatttcatatccttccgcatccaatagattttccatctatccagagaacgtatgtacttgattcttgctgatggcaggatgtttgtttcacctggattgccctgccacacggggcgctgttcgccgatatctatcgtttggtcattgatgctataaacaatatccagttcattgcggatatgttcaggcggccttatgctttcaatgaattggtgaacttctttttttactgcttgatattcaaggtcattgaacgccatctatcctccttacccaaacgtctcttcaggccactggttaccagctatgtgacgatgaagtcacgaacttttcagccactcccttgcctcgatgtcatccagatggcgagattgcttcagaataccagctacatactccacctttgctacttgatgataaggcaacgttataggcctgtgatcctggttaatgcttgtaaattggtattctccatctctgtcatagccaagaaccttaatcatgttgtgtccttcaacggttctgacaaacacctcatcacccgggaatactttggtgttaggctcaatgagtacatattctcctgattttattctgggccacatgctgtctcctttcacacgaagaccaaaggcatctggatcatcgctataaattttgagccacccatcgcgctcttcggtcatctcgatggcaccatcaacaccaagaattgcctcaccaaccacgcgcactaaccctttttttaatttgccaacaaatgaaagagtatcttcatcattcgctccatttaacgaagtgccgtgctgaagccaaacaacatcaacgtttagaaatttcgcaagcgcattcattttttcctgacgcggtaaagactcagcattaaaccatttgctaacgcctttggacgaaagagaaagggcacgggctatagccattcccctaccatgttcatcaagaccagcttctttacaggcttgcgctagccgctgggcgaattctttgcgcactttttcattctgaaccatgagtacgatactaaagcacttgcaaaaactttcagttcaaccataatacgtactgaaagtacgaaaaaggatattcctatgcaaaatcttgatgagccgattaaaggtgtcggcatccctgaagttgcgaaggcttgtggagttagcgaaagggctgtctataagtggctcaaaaacggcttcctccctaagactgagttttttgggaaaactaaatacgcatcaaaaatcgaagagatttctggtggcaaatatcaagcaagcgaaatgcttgaaataagcaaaaagaaccttctggctgcataagtaacaccgctattttcacaatggacattcgtcctacgtcgctgacaaagcgagtcccaatatatctgaccaactaaggccatatgcgtttccacgcatacctttcaactagctattcactattggaaatcttaagaaatggaacaaacaagttacagcaaactatcacagcgagaaattgatcgcgctgaaactgatttactcatcaacctgtcaacgcttacccagcgcggtctggcaaagatgattggctgtcatgaatcgaagataagcagaacagactggaggtttatagcttcggtcttgtgtgcttttggcatggcatcagacatcagtccgattagcagagcttttaagtatgcgcttgatgaaatcacaaagaaaaaatccccggtggccgccggggactctaagcaaattgatatgcaattctgagggaattactggatcaatccacaggagtcattatgacaaatacagcaaaaatactcaacttcggcagaggtaactttgccgaacaggagcgtaatgtggcagatctcgatgatggttacgccagactatcaaatatgctgattgaggcttattcaggcgcagatctgaccaagcgacagtttaaagtgctgcttgccattctgcgtaaaacctatgggtggaataaaccaatggacagaatcaccgattctcaacttagcgagattacaaagttacctgtcaaacggtgcaatgaagccaagttagaactcgtcagaatgaatattatcaagcagcaaggcggcatgtttggaccaaataaaaacatctcagaatggtgcatccctcaaaacgagggaggttcccctaaaatgagggacatccctcaaaacgagggaaaatcccctaaaacgagggataaaacatccctcaaattaggggattgctatccctcaaaacagggggacacaaaagacactattacaaaagaaaaaagaaaagattattcgtccgagaattctggcgaatcctctgaccagccagaaaacgatctttctgtggttaaaccggatgctgcaattcagagcggcagcaagtggggaacagcagaagacctgaccgccgcagagtggatgtttgacatggtgaagaccatcgcaccatcagccagaaaaccgaattttgcagggtgggctaacgatatccgcctgatgcgtgaacgtgacggacgtaaccaccgcgacatgtgcgtgctgttccgctgggcatgccaggacaacttctggtccggtaacgtgctaagtccggccaaactccgcgacaagtggacccaactcgaaatcaaccgtaacaagcaacaggctggcgtgacagctggaaaaccaaaactcgacctgacaaacactgactggatttacggggtggatttatgaaaaacatcgccgcacagatggttaactttgaccgtgagcagatgcgtcggatcaccaacaacatgccggaacagtacgacgaaaagccgcaggtacaacaggtagcgcagatcatcaacggtgtgttcagccagttactggcaactttcccggcgagtctggctaaccgggaccagaacgaactgaatgaaatccgccgccagtgggttctggctttccgggaaaacgggatcacctcgatggaacaggttaacgcaggaatgcgcgtagcccgtcggcagaatcgaccatttcttccatcacccgggcagtttgttgcatggtgccgggaagaagcatccgttatcgccggactgccaaacgtcagcgagctggttgatatggtttacgagtattgccggaagcgaggcctgtatccggatgcagagtcttatccgtggaaatcgaacgcgcactactggctggttaccaacctgtaccagaacatgcgggccaatgcgctgactgacgcggaattacgacgcaaggctgccgatgaactgacctgtatgacagcgcgaattaaccgtggtgagacgatacctgaaccagtaaaacaacttcctgtcatgggcggtagacctctaaatcgtgcacaggctctggcgaagatcgcagaaatcaaagctaagttcggactgaaaggagcaagtgtatgacgggcaaagaggcaattattcattacctggggacgcataatagcttctgtgcgccggacgttgccgcgctaacaggcgcaacagtaaccagcataaatcaggccgcggctaaaatggcacgggcaggtcttctggttatcgaaggtaaggtctggcgaacggtgtattaccggtttgctaccagggaagaacgggaaggaaagatgagcacgaacctggtttttaaggagtgtcgccagagtgccgcgatgaaacgggtattggcggtatatggagttaaaagatga |
| Region of P22 phage in λ*imm*P22dis phage (blue text) flanked by the *J* and *ninB* genes of λ (black text). | atgggtaaaggaagcagtaaggggcataccccgcgcgaagcgaaggacaacctgaagtccacgcagttgctgagtgtgatcgatgccatcagcgaagggccgattgaaggtccggtggatggcttaaaaagcgtgctgctgaacagtacgccggtgctggacactgaggggaataccaacatatccggtgtcacggtggtgttccgggctggtgagcaggagcagactccgccggagggatttgaatcctccggctccgagacggtgctgggtacggaagtgaaatatgacacgccgatcacccgcaccattacgtctgcaaacatcgaccgtctgcgctttaccttcggtgtacaggcactggtggaaaccacctcaaagggtgacaggaatccgtcggaagtccgcctgctggttcagatacaacgtaacggtggctgggtgacggaaaaagacatcaccattaagggcaaaaccacctcgcagtatctggcctcggtggtgatgggtaacctgccgccgcgcccgtttaatatccggatgcgcaggatgacgccggacagcaccacagaccagctgcagaacaaaacgctctggtcgtcatacactgaaatcatcgatgtgaaacagtgctacccgaacacggcactggtcggcgtgcaggtggactcggagcagttcggcagccagcaggtgagccgtaattatcatctgcgcgggcgtattctgcaggtgccgtcgaactataacccgcagacgcggcaatacagcggtatctgggacggaacgtttaaaccggcatacagcaacaacatggcctggtgtctgtgggatatgctgacccatccgcgctacggcatggggaaacgtcttggtgcggcggatgtggataaatgggcgctgtatgtcatcggccagtactgcgaccagtcagtgccggacggctttggcggcacggagccgcgcatcacctgtaatgcgtacctgaccacacagcgtaaggcgtgggatgtgctcagcgatttctgctcggcgatgcgctgtatgccggtatggaacgggcagacgctgacgttcgtgcaggaccgaccgtcggataagacgtggacctataaccgcagtaatgtggtgatgccggatgatggcgcgccgttccgctacagcttcagcgccctgaaggaccgccataatgccgttgaggtgaactggattgacccgaacaacggctgggagacggcgacagagcttgttgaagatacgcaggccattgcccgttacggtcgtaatgttacgaagatggatgcctttggctgtaccagccgggggcaggcacaccgcgccgggctgtggctgattaaaacagaactgctggaaacgcagaccgtggatttcagcgtcggcgcagaagggcttcgccatgtaccgggcgatgttattgaaatctgcgatgatgactatgccggtatcagcaccggtggtcgtgtgctggcggtgaacagccagacccggacgctgacgctcgaccgtgaaatcacgctgccatcctccggtaccgcgctgataagcctggttgacggaagtggcaatccggtcagcgtggaggttcagtccgtcaccgacggcgtgaaggtaaaagtgagccgtgttcctgacggtgttgctgaatacagcgtatgggagctgaagctgccgacgctgcgccagcgactgttccgctgcgtgagtatccgtgagaacgacgacggcacgtatgccatcaccgccgtgcagcatgtgccggaaaaagaggccatcgtggataacggggcgcactttgacggcgaacagagtggcacggtgaatggtgtcacgccgccagcggtgcagcacctgaccgcagaagtcactgcagacagcggggaatatcaggtgctggcgcgatgggacacaccgaaggtggtgaagggcgtgagtttcctgctccgtctgaccgtaacagcggacgacggcagtgagcggctggtcagcacggcccggacgacggaaaccacataccgcttcacgcaactggcgctggggaactacaggctgacagtccgggcggtaaatgcgtgggggcagcagggcgatccggcgtcggtatcgttccggattgccgcaccggcagcaccgtcgaggattgagctgacgccgggctattttcagataaccgccacgccgcatcttgccgtttatgacccgacggtacagtttgagttctggttctcggaaaagcagattgcggatatcagacaggttgaaaccagcacgcgttatcttggtacggcgctgtactggatagccgccagtatcaatatcaaaccgggccatgattattacttttatatccgcagtgtgaacaccgttggcaaatcggcattcgtggaggccgtcggtcgggcgagcgatgatgcggaaggttacctggattttttcaaaggcaagataaccgaatcccatctcggcaaggagctgctggaaaaagtcgagctgacggaggataacgccagcagactggaggagttttcgaaagagtggaaggatgccagtgataagtggaatgccatgtgggctgtcaaaattgagcagaccaaagacggcaaacattatgtcgcgggtattggcctcagcatggaggacacggaggaaggcaaactgagccagtttctggttgccgccaatcgtatcgcatttattgacccggcaaacgggaatgaaacgccgatgtttgtggcgcagggcaaccagatattcatgaacgacgtgttcctgaagcgcctgacggcccccaccattaccagcggcggcaatcctccggccttttccctgacaccggacggaaagctgaccgctaaaaatgcggatatcagtggcagtgtgaatgcgaactccgggacgctcagtaatgtgacgatagctgaaaactgtacgataaacggtacgctgagggcggaaaaaatcgtcggggacattgtaaaggcggcgagcgcggcttttccgcgccagcgtgaaagcagtgtggactggccgtcaggtacccgtactgtcaccgtgaccgatgaccatccttttgatcgccagatagtggtgcttccgctgacgtttcgcggaagtaagcgtactgtcagcggcaggacaacgtattcgatgtgttatctgaaagtactgatgaacggtgcggtgatttatgatggcgcggcgaacgaggcggtacaggtgttctcccgtattgttgacatgccagcgggtcggggaaacgtgatcctgacgttcacgcttacgtccacacggcattcggcagatattccgccgtatacgtttgccagcgatgtgcaggttatggtgattaagaaacaggcgctgggcatcagcgtggtctgagtgtgttacagaggttcgtccgggaacgggcgttttattataaaacagtgagaggtgaacgatgcgtaatgtgtgtattgccgttgctgtctttgccgcacttgcggtgacagtcactccggcccgtgcggaaggtggacatggtacgtttacggtgggctattttcaagtgaaaccgggtacattgccgtcgttgtcgggcggggataccggtgtgagtcatctgaaagggattaacgtgaagtaccgttatgagctgacggacagtgtgggggtgatggcttccctggggttcgccgcgtcgaaaaagagcagcacagtgatgaccggggaggatacgtttcactatgagagcctgcgtggacgttatgtgagcgtgatggccggaccggttttacaaatcagtaagcaggtcagtgcgtacgccatggccggagtggctcacagtcggtggtccggcagtacaatggattaccgtaagacggaaatcactcccgggtatatgaaagagacgaccactgccagggacgaaagtgcaatgcggcatacctcagtggcgtggagtgcaggtataccatgaaaaaagttaacattggaaacgtaccaaagatgctcgttccgctctttgagagcggtacaattgtgttttgtagagactttccagaatggcaacgcctgcatcaaaaacttggtgtggacgtgcaggactcggatgccaacggagcgtctcatacaatgagcagcgagaatggtgttttgcatgtgataggcgtgttcaatggcaaactatctactattgcccatgagtgcgctcacatggcattcgatatctgctcaagggtcggtgttgatgttgaaccaggaagagccaacgagacttactgctacttaatgagcaggcttgttgagttctgcgagcgacatatcaaaaagccggagtgacccggcttgattattactttttgctgtctggagttcgcttatctaatacccagccattacctggctttgttgttggtggaagcctttcgttgtccttgacggtggcaaaattgtctttcttaccgcctcgcgggccaacttcttggtatattccgccgttttttcctgtgttttcacctggttttttcgccatgatatacctcaacatacacccgttattgggcgattaaatattgatctcattttataagtagtcaatatggcccaggtaaatgcaaaaattaacccgccgtcaggtggtttttttgtacaaatccttcagcgtatcaaacaccatcttcttaacaagctctgactgctcatcagcgagtcgttctgcatcgttgcgatatccagtcacaggcgatggttttgatagagcatcttggacgatttgtaacaactcggagttcattgatctcccattcgcctccgccctgaattttaatttctccctgacttccataggcatacggaagttaaagtgcggatcatctctagccatgccatcactccaagttagtgtattgacatgatagaagcactctactatattctcaataggtccacggtggacctgtattgtgaggtgaatatgaaaggaatgagcaaaatgccgcagttcaatttgcggtggcctagagaagtattggatttggtacgcaaggtagcggaagagaatggtcggtctgttaattctgagatttatcagcgagtaatggaaagctttaagaaggaagggcgcattggcgcgtaaagttgaagccccaactgcggtaacagtcagggcttcggttgtcagtaaatccttggagaaaaaccaacatgaatagtatagcaattttagaagcagttaacacctcttacgtgccgtttaatggacagcatgttcttaccgctatggtggctggagttgcctatgtagctatgaagccagtcgtggataacattggtctctcatggtcatctcaggtgcaaaagcttctgaaaatgaaagataaattcaactatgtcgatatcgacatggttgctggagatatgaagaaacgtctcatgggatgcatcccactgaagaaacttaacggctggctgttcagcattaaccctgagaaagttcgtgcagacatccgtgacaaactgattaagtaccaggaagaatgcttcaccgttctgtatgattactggacgaaaggtaaggctgaaaacccgcgtaagaaaacatctgtcgatgagaggacgccgcttcgtgatgctgtaaatatgcttgtaagcaaaaagcatctgatgtacccagaagcttatgcaatgatccatcagcgtttcaatgtggaaagtattgaagaactggaggcgtctcagataccgctggccgtagagtacatccacagggtagtgcttgaaggtgagttcattggcaaacaagagaagaaaaccaacgatctttctgcaaaagaagcaaacagccttgtatggttatgggattatgccaaccgctcacaggcgttattccgcgaactgtatcctgcaatgagacagattcaatctaactattcaggaaagtgctacgactacggccatgaattctcgtacatcattggaatagcgagagacgttttaattaatcacacgcgagatgttgatattaatgaacctgacgggccaacgaatctttccgcatggatgagacttaaggataaagagcttccaccttcattacatcgctactgacagataaccaacgcaacgacccagcttcggctgggtttttttatgcccaaaattcaccgtagccacgcttaggtaatgagcttgaaggagagacctacaaaaaaattgtaggtcgaaaagcgaacaaaataacttccgaaaaagttgttttatcacaaaaaattcaccgtagccatgctgcggcaattccttgcatctggagcaaattaaatgacagacatcactgcaaacgtagttgtttctaaccctcgtccaatcttcactgaatcccgttcgtttaaagctgttgctaatgggaaaatttacattggtcagattgataccgatccggttaatcctgccaatcagatacccgtatacattgaaaatgaggatggctctcacgtccagattactcagccgctaattatcaacgcagccggtaaaatcgtatacaacggccaactggtgaaaattgtcaccgttcagggtcatagcatggctatctatgatgccaatggttctcaggttgactatattgctaacgtattgaagtacgatccagatcaatattcaatagaagctgataaaaaatttaagtattcagtaaaattatcagattatccaacattgcaggatgcagcatctgctgcggttgatggccttcttatcgatcgagattataatttttatggtggagagacagttgattttggcggaaaggttctgactatagaatgtaaagctaaatttataggagatggaaatcttatttttacgaaattaggcaaaggttcccgcattgccggggtttttatggaaagcactacaacaccatgggttatcaagccttggacggatgacaatcagtggctaacggatgccgcagcggtcgttgccactttaaaacaatctaaaactgatgggtatcagccaaccgtaagcgattacgttaaattcccaggaatagaaacgttactcccacctaatgcaaaagggcaaaacataacgtctacgttagaaattagagaatgtataggggtcgaagttcatcgggctagcggtctaatggctggttttttgtttagagggtgtcacttctgcaagatggtagacgccaataatccaagcggaggtaaagatggcattataaccttcgaaaaccttagcggcgattgggggaagggtaactatgtcattggcggacgaaccagctatgggtcagtaagtagcgcccagtttttacgtaataatggtggctttgaacgtgatggtggagttattgggtttacttcatatcgcgctggggagagtggcgttaaaacttggcaaggtactgtgggctcgacaacctctcgcaactataatctgcaattccgcgactcggtcgttatttaccccgtatgggacggattcgatttaggtgctgacactgacatgaatccggagttggacaggccaggggactaccctataacccaatacccactgcatcagttacccctaaatcacctgattgataatcttctggttcgcggggcgttaggtgtaggttttggtatggatggtaagggcatgtatgtgtctaatattaccgtagaagattgcgctgggtctggcgcgtacctactcacccacgaatcagtatttaccaatatagccataattgacaccaatactaaggatttccaggcgaatcagatttatatatctggggcttgccgtgtgaacggtttacgtttaattgggatccgctcaaccgatgggcagagtctaaccatagacgcccctaactctaccgtaagcggtataaccgggatggtagacccctctagaattaatgttgctaatttggcagaagaagggttaggtaatatccgcgctaatagtttcggctatgatagcgcagcgattaaactgcggattcataagttatcaaagacattagatagcggagcattgtactcccacattaacgggggggccggttctggctcagcgtatactcaacttactgctatttcaggtagcacacctgacgctgtatcattaaaagttaaccacaaagattgcaggggggcagagataccatttgttcctgacatcgcgtcagatgattttataaaggattcctcatgttttttgccatattgggaaaataattctacttctttaaaggctttagtgaaaaaacccaatggagaattagttagattaaccttggcaacactttagatatgtaataaaaatgggtgtaaacacccatttttattttatgttaaatattctatagctaattaaacctaacaactatggtttcccctacaacaccaatatcgtatacgttattaccagattttttccacccattttcaagtttaacctctttgtcatatagtctgtaatttctggaaaacacatttctttgcattaacacctctgaccacatccaatcattgttaataatgcgtggtattaactctctcattaaaggatgctttattactatgttttcatttattggtgcatacggttctgtgccaatgaattttatattttttttgtctcttccaaatccaagataatctatgtcttgagatattctatttacaatgctttcctcaagctgaaactgtgcatttatggcattgtaagcaccataagaaaatattgttgatattaaaagaataaaagaaaaatatattcttgatattaactgtttatcttcaaaagcatagaatacgcataggcaacaaaaaaacataaagccacccataccaatcaataccctcggtgcgtatattggtgattttagaaaaatcattggtccaatgatgaagaacattgatgctaataaaattaaaactactagcaataactttgttttcttattttcatctcttttgattgcttttaaaactatgactatcaaagaaatgattagcgcaaagaatagcgagtagtagattaagtaattatcgccattcaagatcgtgctaaacattctataaaatgataagacgttagaaattatcccttcaaataaacttgagtttatctctataatcttactatgttcgatattgtaagaacctgttacaagtctttttgcaataaagtaagaataggcaaaatatcctactattaaaccagcgacagaagatgctgtattttttgtgatatttgaaattgagtttttcttaaccacatctgaaattataaaggccaacaagaatattgcgtaagtattcagcgcagcctgataaagactaaggaatgcaatggttaaaatggatgatattatgatatttataggcttgtattgataagcgacatacgatgagataatagatattgccacactcatgcacattgttaatgaatcatatctatatgatagattttcaataaagaatgggtttgccaaaatcatcataaaacaaagagatgctgtgatgtagtcatctccaaacagcttttccctgatgcaggatagtgccaatgctaaaataactatccctagcattaaaggtagcggagaagcatctataattggggttccaaaattaatgatatagaaaataaagtcggaaagtgggcgaccattgcctgaccaacccaacccgccatataaagacctacccaagtcatcaacgaaaaatgattgatgtgtcaataaaggaaatgtatatataatcgccaatccaagaaagattgatataaatatcctgtcattactattaaatttcacttttaaaacccttacgctttaatatgtatttaggccgctgtttggtttctatgtaaattctaccaatatattctccaagaatacctattcctatcaattgaacgccacccaggaaaagaacagaaacaagaagagacgggtagccaggaacattatttccaaatattaatttatcaataatcatccatgcaccgtaaaggaatgacatacctgcaataaacaatccaatgtaagtccatatgcggagcggaaatgttgagaaagaagttattccctccagcgccaggttccataatttccagccgttgaatttcgaatcaccggccacgcgttcggcacgggcatatttaacaacatccgtttttccgccaacccaactgagcacacccttcataaacaagttgcgttctggcatttgtttgatgttctcgacaaccgcacggctcattaaccgaaagtcgccaacattttcttcgatttttggattgctgattttattgtgcagcttataaaaccactcagctgtcttacgcttcatgcgcccgtcagttgagcggtctgagcgcttagccagcaccatatccgcgccagcctgccacttctcaatgagatgagggataacttctatcggatcctgtaaatcgacatcaataggaatgaccgcatccccggttgcatggtcgagacccgcgaaaagagcaggttctttaccgaagtttcgcgtaaacgaaagcggaataacgagcggatcagatgcagctattttgttaattattgattcagtcgcatctttactaccatcattaataaaaacgatctcaatttcatattcttttagctcattaaactcacgtaccgttttatagaaaatcggtatcgtgtcttcttcgttaaaaactggaacgacaagagagattttcatcttatatccctgaaaacaatgaatctggaatagataaagccgcataccaggctaattgccgagaaagtgataagggtaatcaatggtggcaaggaacattggtcagccatccagccaacaacagcgctcagtgttcccatgaatcccacacacatcatgtagcgaagcgtggtggtggtggcattaaaggtgaaacgcgcattggcatagaagctgaacgatacggcgataacaaaaccggaaaagttcgccagcgcctgatgcgtatgcatcccatacacacaaaaagcaaatacgccccaatgaataagcgtgttaagaacaccgatcgatgtgtacttagcgaataacttcaacattatgaaaatcagcggattcggaaaggtctgaagtgtagcactacaaattgttttgatcgatacaagcgatcaataatgtataatttgatagtttttatctatataatgcatgttaattgatcgttgttaccgatcaatttttattgctgattgctaagtggtttgggacaaaaatgggacatacaaatctttgcatcggtttgcaaggctttgcatgtctttcgaagatgggacgtgtgagcgcaggtatgacgtggtatgttgttgacttaaaaggtagttcttataattcgtaataataagttcggctggtagaatgtcgggattgtatgcaagtcctctcatcgtaaactcctcagttattgctgatagctccgtaacgcgaacggtaatcacgaagacgcgggtctatttcaatgaatttggtgtaagtggctttgcggaatggccggatggctgtctggtaaattcgctcgcgttcttctttctctgcaagccatatacagtggcgaaattccttttcctctttcgtttcctgcggtagtgacattatcaggtcgtagttttttctgaatttatccagcacctccgatacggaattgccggaacagcggcgcgggtcatccgcaccatacaaaggcgctggcataatttactccagggtaggttatccgaataatgtggtacgtatagggttatttctttcgtaaacgtgatagcctgctttttaccgactcttcacttcgcccgagaatttttgctacatttctttgtgtatagcctgatgagataagcgtctgcattcttttgtcttcgtcgtcgctccatcttggcttaatgaatgccgtttttaatgacagtttttttgctatgtaataaaactgatttatgtttaggcccagatgttctgctgcacggcaagctaccatgcgaccgcaaactgactccatctccgctgtagttatgtttaatcttctcattaagccacctgtttaagctcatttattctgatattcattacctgaacgcattttgtctgctcatcatcgtgaccagtcaataattgccagtcgtgctggtatctctcaattagctttttcttgtccgtttctgttgctgcatattcactgaatgccttaagaacctgttcaggagcaggggaggaaggcgatgatttagtttgcttagcaggtgctgcattctgctgctgtttgtgctcctcagtatcagcgtctttggcgtcgtcgataccaaacaaaccgttaaggcaatatttgcgagcgtaagagcttgtagcgcccgttacctgagctgcatccattcccttcttgttttcttcttctcgcgctatagcgcttgctgaatggctattttcaccatctgtaatggtcgctgtggccttgacgtaataacggtcgccaatcagcacgatttcatcactgatagacaggaacagacctttcagtagtggcttaacaccctcaagaatgtcctcacaactgcggtatttgtatttaccaaacgagttgtactgattctttggcgcattcagatgctcctgaatttcagcaagtcttgcgtaaaactctttgctcatgagtaataccccgcaaattcatcccaaccaataatcggattttgccgttctgcggctaagttgatttgttgctcaacctcttcctcaatttcaggagagataagcgcgataaattcttcgtcactaaattcatgctgcatgatttcgattccagtcttcgtcctgacaatcttcccagcccattgcgattgatgatgcccatgcgtatgcggcgctgttaccttccttcgtatccgggaaggatgcttcgtagagcttgttaaactcacgattgccttgctgcacaaggatggttccgttaacaggcacaatagtcatggctcggcactccaggctgattaaggatgtctgccagccgtttccagccagcgcgtaatttgcgggtgatgcgatctaaaagtgattcgtgtaactgggaaacgcccatgcgagcgcttcccgcgattgcgataatcatgggagttccttatgttgtgtgtgattgcatgaggctgagcacttgaataaatactcactcagatgcggatatgaaaaagccgcactcaggcggctgtcgtttcttctttcaggctttcgagatattcacgcgggtcgtcgtaacactggcactcgctataccaatcaatccagcgatcatccagttccatatcttccaaatcctggtcggtaaggctctcgtcaaacatctgtaaaccgttggcgttgcagtaatcaggcttgatgttgttgtcatactgaaaggcgtcataatcagccagtgcatccatcattcgtacaccttcttcaacactacccacttctacaatgaatggcttcatagggacttgcgggatatgccagacacgtaatttcatatttcctccaggcaaaaagaatgccgcccatatagagcggcaagactatcaagggatgattctccaataaccagaacgagtcttcgtcctcattcggttacgagcgatattgctcacatagcagactcgtaaatctgctataggtgcttattcgcttggtggttcaggtaatggcatccagtgagtcactcgctcatgcttgattaaacgcgtgtcctgctttgagttctttgcctcaattactggcttcctcatggtgtaaattcccgagaaatttgaattgcaaacacaatatccaagcacagtaattccggattccggcatccgctcactacacttaatccactccatcactccttccccagagccttgccgatggctgcgcgagccgtcgcgtacacagcgtcccactcgctcacgtcatgttccatatcgataatgcttaacagagcctcgagaagctcaggagctgcacttgccaattttatttcctgcttattggcgttctcggctgaaaacagaatgtgaccttcagtaacatcgacaatgtaatcttcatcaacatcccaatgcggtatgtctttcatattcacctctgtggcttgctgccaaaagaagacagactatatagcctttagtttttccagctctctggcaatcatttccgtggttctgattgcccatttatcgacaatctttccatcttctctcaccagagccatttcctcaggctttaccatacattcagcatcaagcttgcagcctttgcatttcacaaaacgactacaccattgatttgtatcaatagtcgtagtcatatgggtagtcctggtattgttccatcacatcctgaggatgctcttcgaactcttcaaattcttcttccatatctcatctcaaatagtggattgcggtagtaaagattgtgcctgtcttttaaccacgtcaggctcggtggttctcgtgtacccctacagcgagaaatcggataaactctattcacccctacagagagtaaaaagagaatcgccgatgaacaactcatggtggcaggagttaatgcgttttttcctgcaaggaatgacacttaaacagttgattcatatgctaatcatcctgatcgtattgattattgttatgccggtaagcgtaaaagaatggataaacctgcataatccagaaatacttcctcattactggatgtattacatcctgttgttctgcgttagctatgtgcttaacggtgttgttaattccgtttatcacgctgttactgaaagaattgaggcatcaactgctcagcggcgtaaggacagagaagaaaaagtcgttcgggatttgtttgattcgttaactcttggagaaagagcgtatttggcattcgctgtagccgctaataaccagctaaagacagaaaagggaagccctgaagcaatttcattgctcaaaaaagggattatcactcgattgccttctgctattggatatcctgatattgaccgttttattatcccggaaaagtattttaatgagtgctacatgagatttgccgggaagtcagacattcttatgaatgaacttattgtacaggacgaacagctcaaaaaataacgacttaaccgacaaataccttacctcgctgttatttgtttgctcttacgatgaccagccgcgtaaagtgctacgcctggaagaagtacagatcctccttcaacttccttctgacgcgttccggcaagcgaaatggctttggtgacacggtcaattcttttggctttaacttcctgagaagcatcaggagcatcgcagccaaaaattgaatcgatgatattgcagatggtgtcgcgctctatggctagctttctgcgccgctcatgacggcgagttttagcattgcctgcaaacgttgacttcccgtaggtgataaccgtcatgatttaatcctcatgtgaaatggctttggtactggcgccggaacctgtctcaatttccggatttcaagtggcttctcagtccggcccgatcggtacagctagaggcctaagctccaccacacgccagtccaaaccaatctcgtttggtatttgttcgcgctttgtcagcgcatcatcgaagttaaagagcgttgcctttccgtttggctaccagcgtcctgctgatggctaaaatttaagacttcttaattaaatggtcaagtgtatttttgaagaaaacttaaatattttatcgttacttaagtttttatttgatttttaaaggaaaatgtagtgtgaggggcgggtgccccttatggaagatttgcgagttttgcgtcaacaactacgccaatgattttgcagtttccgttgatttctatcatcggatattgtgggtttaatggttttaaaaactttcggcctgcatccataactaattttttgaatgtggcctcgttttcaccttctaattttgcaacaaccagcttgccgtttcttggttcgacttcgggatcaaccagaattatcattccttctggaatgcttaaccctgccggtgctgtcatagagtcaccttggacatcaagccaaaatgaatcttctgaacaatctacagtggtgtcgtgccagttctctatcgcgcgcttgtgataaggttctacagcttccatccattgccctgcgcttacccaactgataagagggtatgatcctcttggctcatgcctactatgataggcaacgtttgtctggcttaaatctcctttcagcaaatagtcaggggagcactgaagagccttcgaaagtgccaacaggttctccccatttggctcagtctccgagcgctcccattgcgatattgcaacattagacactcccaccatcttaccaagagcggcttgtctaatcttgagtttttttcttcgagcgcgaatacgctcacccatcaattgtgtattcatagttaagtcatcttaaataaacttgactaaagattcctttagtagataatttaagtgttctttaatttcggagcgagtctatgtacaagaaagatgttatcgaccacttcggaacccagcgtgcagtagctaaggctttaggcattagcgatgcagcggtctctcagtggaaggaagttatcccagagaaagacgcataccgattagagatcgttacagctggcgccctgaagtaccaagaaaacgcttatcgccaagcggcgtaagcaaaacgctctttaccaatctgaaccgccgacaacgcggtaaacctatttcaaagcgcatcaacgaatgcgcacaactaactattaactacaggaatgttcacatatggaactcacaagcactcgcaagaaagccaacgcaattaccagcagcatccttaaccggatagctattcgtggacagcgtaaagtcgctgatgcgttaggcattaacgaatctcaaatttcacgatggaaaggcgatttcattccgaagatggggatgttattggcggttctggagtggggtgtcgaggatgaggagttggcagaactggcaaagaaagttgcgcatctgctgacaaaagaaaagcctcaagactgcgggaacagttttgaggcctgatgtagaaagactggatcaatccacaggagtaattatgccaaaacaactcagtcctgaccaggacaaattacacaaaaacatactacgtgatcggttcttatccagcttcaaacagcctggtcgatttcgggctgagttggagaaagtgaagctaatactgaagaggaaaggtcatgagtaatcttgcaacagttacaccgataaaacctcatctggaggttgtggagcatcgcgtggcagaactcgacgatggctacacccggactgcaaatacactgctggaagctgtcatgctttctgggcttactcaacatcagctactgattgttatggctgtgtggcgcaagacatacggttataacaaaaaaatagattggatcggaaatgaacagttcgctgaactcactggcatggcgccaaccaaatgttctaccgccaaaaacgagcttatcagaatgggggttctcactcaggtggggcgtcaggttggtatgaataaaaatatttccgagtggaagacgaaggttaacggattcggtaaaacatttaccagatcggtaaaactaaccttcaccaaatcggtaaaaaccaatttaccgaatcagtcaaacacaaaagacaatatacaaaagacaataaatacaaatacccccttaccccctaaagggggatgcgatgaaggttctaaacctgaaaagcgaaaacctaccaagattaactacagcgaatatcttgctgcctacaacgagattgttggtgacagactcccacatgcagtggaggtcaattctgaacgacaacgcaagttgaaaaagctgattgattcactggcaaccaaaaacatcgacggattccgggcatacgtcaaagcgttcatggcagcagccagaccattccatttcggtgataacgaccgtgactgggtagctaattttgattatctgctacgcccgaaagtactgatagcaattcgtgagggaacactatgagacaggatatcgaggcgagcgttatcggtggcttgctgattggcggattaacaccaaccgccagtgacgttctggcaacactggagcctgaagcattctcaattccgctctaccggaaagcttttgaagttattcgaaagcaggccagaaacaggaacctgattgatggactgatggtggccgaggagtgcggggatgaatacgcaacggcggtgatgatgactgcgcggtcatgtcccagcgctgcaaacctgaaaggttatgccggaatggttgcagacagttatcaacggcgtcaggttttacagctactggatgagatgcgggagccaatcagtaacggcacgctggacgcatcaggcagagcgatggacgagcttgtaaagcgcctgtcatccatcaggaagccgcggaacgaggttaaacctgtgcgactgggtgaaatcatcaatgactacactgacacgcttgacaggcgtctgaggaacggagaagagtcggataccctgaagaccggaatcgaagagcttgacgctatcaccggagggatgaacgcagaagaccttgtgattattgctgctcgtccaggtatgggtaaaaccgaactggcgctgaagatagccgaaggcgtggcaagtcgtgttattcctggttctggcgtccggcgcggtgtgttgattttctcgatggaaatgagcgccattcaggttgttgagagagggattgccggcgcaggaatgatgtcggtcagtgtgctgcgtaacccgtcacgtatggacgatgaaggatgggcgagagttgcaagcgggatgaagttgctggcagagctggatgtgtgggtagttgacgcatcgcgtttgtctgtcgaagaaatcaggtccatttccgaacgccacaagcaggagcatcctaatctgtcactgattatggctgactatctcgggctaattgagaaaccaaaagcggaacgtaatgacctcgccatagcacatatctccggtagcctgaaagcgatggcgaaagacctgaaaactccagttatctccctaagccagctctcccgcgatgttgagaagcggccaaacaagcgcccgacaaacgcagatttgcgggattcaggaagcattgaacaggacgcagactcaatcatcatgctctatcgggaagcggtatatgacgagaacagtagcgccgcgccatttgctgaaatcatcgtgacgaaaaaccgttttggctcgcttggtacggtttaccagcggttctgcaacggacactttgttgcatgtgaccaggacgaagccagacagatttgcacggcatcaaatgcacctgctggacgcagaaagcgatatgcacaaggggctgacgtatgactatttacatcactgagttggtaacaggcctgctggtaatcgcaggcctttttatttgggggagagggaagtcatgaaaaaactaacctttgaaattcgatctccagcacatcagcaaaacgctattcacgcagtacagcaaatccttccagacccaaccaaaccaatcgtagtaaccattcaggaacgcaaccgcagcttagaccaaaacaggaagctatgggcctgcttaggtgacgtctctcgtcaggttgaatggcatggtcgctggctggatgcagaaagctggaagtgtgtgtttaccgcagcattaaagcagcaggatgttgttcctaaccttgccgggaatggctttgtggtaataggccagtcaaccagcaggatgcgtgtaggcgaatttgcggagctattagagcttatacaggcattcggtacagagcgtggcgttaagtggtcagacgaagcgagactggctctggagtggaaagcgagatggggagacagggctgcatga |
| Region of 21 phage in λ*imm*21 phage (blue text) flanked by the *orf28* and *O* genes of λ (black text). | tcaatagtcgtagtcatacggatagtcctggtattgttccatcacatcctgaggatgctcttcgaactcttcaaattcttcttccatatatcacctcaaataagtggtttgctgcctaatttaattttctggcgaccaacacaagtcacacccatttcactgcgtggcttgctgtagtaaatacggttctgtttacgctcgacttcttctgccttcttgcagcgaaggcttccgagtgatgctgctttatctgctctgacgcaaccagagagctttagcgcaatttttcgcgccagtgcttcattactgcgtcgctcggcaataagttctgctctgcgagctttgtagcggctttttgccgtacctttggattctttccagacaatggttaccatgatggtctcctttaagtggctttggcgcatgacgcgtcgaggtgcttatcttctcgatcgctgtcttgtagctgcaattcgcgccatccccaaaaccactcaagttctggtctcaacggttaggttgagagtccgtcgatgttaaagagcctgccaatctgttccgtttggcttccagcgtcctgctgatggcttaaatttaagacttcttaatttattggtcaagtgcatttttgaagaaaacttaattttatgggcgtgaatttagtttgtctttgatttttaacgggaaataaaaaaggggcgaaagccccttaaggaaggtttgctagcttggcatcaacgacaacgccaatgattttacagttcccattgatttcaatcattgggtattgtggattgagtggtttcaggaattttctaccggcatcaataactaactttttgaatgtcgcctcgttttctccttcaagtttggcgactaccagctttccattacgtggttcgacttctgggtcgacgagaataatcatcccctcaggaatactcagtcctgccggggcagtcattgaatcgcctttaacgtcgagccaaaaagagtcttcagaacaatctaccgttgtgtcgtaccagttatctattgcacgcctatgatatggctctacagcttccatccaacatcctgcgcttacccaactaattagaggatacgaacctcttggatcatgcctgctgtgataggcaatgtttgaaagactatcctctcctttcaacaggtaatcaggggagcactgcaaagccttggctaaggccaataggttttcgccattgggctcagtttcagatcgctcccattgggaaatagcaacattagacacgccaaccatcttgccaagggcagcctgcctaatcttgagttcttttctgcgagcgcgaatacgctcacccatcagttgtgtattcatagttaagacatcttaaataaacttgacttaagattcctttggtggataatttaagtgttctttaatttcggagcgagtctatgtacaaaaaagatgttattgaccacttcggaacccagcgtgctgttgctaaagcactaggcattagcgatgcagcagtctctcagtggaaagaagttatcccagagaaagacgcctatcgattggaaatcgttacagctggcgccctgaagtatcaagaaagtgcttaccgccaagcggcataagcaaattgctctttaacagttctggcctttcacctctaaccgggtgagcaaacatcagcggcaaatccattgggtgtgccgctataactcaatatcaatataggtaaattaacaaatggcacaagcaagctacagcaagccaacacagcgagaaattgatcgcgctgaaactgatttactcatcaacctgtcaacgcttacccagcgcggtctggcaaagatgattggctgtcatgaatcgaagataagcagaacggactggagatttattgcttcggtcttgtgtgctttcggaatggcatcagacatcagtccgattagcagggcttttaagtatgcgcttgatggactcacaaagaaaaaacgcccggtgtgcaagaccgagcgttctgaacaaatccagatggagttctgaggtcattactggatctatcaacaggagtcattatgacaaatacagcaaaaatactcaacttcggcagaggtaactttgccggacaggagcgtaatgtggcagatctcgatgatggttacgccagactatcaaatatgctgcttgaggcttattcgggcgcagatctgaccaagcgacagtttaaagtgctgcttgccattctgcgtaaaacctatgggtggaataaaccaatggacagaatcaccgattctcaacttagcgagattacaaagttacctgtcaaacggtgcaatgaagccaagttagaactcgtcagaatgaatattatcaagcagcaaggcggcatgtttggaccaaataaaaacatctcagaatggtgtatccctcaaaacgagggaaaatcccctaaaacgagggataaaacatccctcaaattgggggattgctatccctcaaaacagggggacacaaaagacactattacaaaagaaaaaagaaaagattattcgtccgagaattctggcgaatcctctgaccagccagaaaacgatctttctgtggttaaaycggatgctgcaattcagagcggcagcaagtggggaacagcagaagacctgaccgccgcagagtggatgtttgacatggtgaagaccatcgcgccatcagccagaaaaccgaattttgctgggtgggctaacgatatccgcctgatgcgtgaacgtgacggacgtaaccaccgcgatatgtgtgtgcttttccgctgggcctgccaggacaacttctggtccggtaacgtgctgagtccggccaaactccgcgacaagtggacccagctcgaaatcaaccgtaacaagcaacaggcaggcgtgacagccagcaaaccaaaactcgacctgacaaacactgactggatttacggggtggatttatga |
| Region of 434 phage in λ*imm*434 phage (blue text) flanked by the *N* and *cII* genes of λ (black text). | atgatcgttatctgggttggacttctgcttttaagcccagataactggcctgaatatgttaatgagagaatcggtattcctcatgtgtggcatgttttcgtctttgctcttgcattttcgctagcaattaatgtgcatcgattatcagctattgccagcgccagatataagcgatttaagctaagaaaacgcattaagatgcaaaacgataaagtgcgatcagtaattcaaaaccttacagaagagcaatctatggttttgtgcgcagcccttaatgaaggcaggaagtatgtggttacatcaaaacaattcccatacattagtgagttgattgagcttggtgtgttgaacaaaactttttcccgatggaatgggaagcatatattattccctattgaggatatttactggactgaattagttgccagctatgatccatataatattgagataaagccaaggccaatatctaagtaactagataagaggaatcgattttcccttaattttctggcgtccactgcatgttatgccgcgttcgccaggcttgctgtaccatgtgcgctgattcttgcgctcaatacgttgcaggttgctttcaatctgtttgtggtattcagccagcactgtaaagtctatcggatttagtgcgctttctactcgtgatttcggtttgcgattcagcgagagaatagggcggttaactggttttgcgcttaccccaaccaacaggggatttgctgctttccattgagcctgtttctctgcgcgacgttcgcggcggcgtgtttgtgcatccatctggattctcctgtcagttagctttggtggtgtgtggcagttgtagtcctgaacgaaaacaccccgcaatggcacattggcagctaatccggattcgcacttccggccaatgcttcgtttcgtatcacacacaccaaagccttctgctttgaatgctgcccttcttcagggcttaatttttaagagcctcaccttcaatggtggtcagtgcgtcctgctgatggcttaaaattacaaggaagattgtatgttgtaaacaataaatattgtaaaaaggggcgtgaaaaacaaactccattgtttttaaacggaaaatagtttgtttttttgttatcgagattgaggtggggattactgattgcaggttccgactacatcaccaacaaaggatttggttgatgtaagttgttgcatacctgggatgttcattactttggagtaaagagcttttttgtctgtagtgattgaccaagtttcaacggttatgcctcctccagactggtattctcctaccatagtgttcgatgacaaagcagtgtatttcatctctggatagacgccagaaactgattcataaactgatgatttatcgccatttattgttacgttgaaaacggaatcttccgtgctgtcttttgtaaactcgtaacgatcgccattcattgccccgtacccgtgcaggtttgtgacaatccagcattcagaattggcgctggtagttaagagtattgagagtagcgccgcaatcctgatcatacgaattttaccctcgcttccacgacaacaccgataatcttgcagttcccgttgataggagtcataggccatgaaggattcaggcctttcaggtacttctgaccgccatctatgaccagtttcttgaatgttgcttcgttcgcgtcagtcagtttggctacaacaaggcttccattcactggctcgcgtccagtatctactaacaccatatgaccttcagggatgctttgacctacaggtgaggtcatggaatcaccttcaaccttcagccagaatccattgcctaataagttaacgtcactgtcataccattcatcaatgtccttgatatcgtagggttcacaagcttcacaccacgaaccagctctaaccatgctaatcaatggatatttccctttgggctcaacgtgcccaacaaatctaacattcgaatcagaggtgccattgagcagccagtcaacacttacgccaagagctgacgcaagttctggtaaaaagcgtggtcgcttagttttaccgttttcgagctgctctatagactgctgggtagtccccaccttttgagcaagttcagcctggttaagtccaagctgaattcttttgctttttaccctggaagaaatactcataagccacctctgttatttacccccaatcttcacaagaaaaactgtatttgacaaacaagatacattgtatgaaaatacaagaaagtttgttgatggaggcgatatgcaaactctttctgaacgcctcaagaagaggcgaattgcgttaaaaatgacgcaaaccgaactggcaaccaaagccggtgttaaacagcaatcaattcaactgattgaagctggagtaaccaagcgaccgcgcttcttgtttgagattgctatggcgcttaactgtgatccggtttggttacagtacggaactaaacgcggtaaagccgcttaagacattcccgctcttacacattccagccctgaaaaagggcatcaaattaaaccacacctatggtgtatgcatttatttgcatacattcaatcaattgttatctaaggaaatacttacatatggttcgtgcaaacaaacgcaacgaggctctacgaatcgagagtgcgttgcttaacaaaatcgcaatgcttggaactgagaagacagcggaagctgtgggcgttgataagtcgcagatcagcaggtggaagagggactggattccaaagttctcaatgctgcttgctgttcttgaatggggggtcgttgacgacgacatggctcgattggcgcgacaagttgctgcgattctcaccaataaaaaacgcccggcggcaaccgagcgttctgaacaaatccagatggagttctga |
| Region of P22 phage in λBH2 phage (blue text) flanked by the *orf28* and *ninB* genes of λ (black text). | tcaatagtcgtagtcatacggatagtcctggtattgttccatcacatcctgaggatgctcttcgaactcttcaaattcttcttccatatctcatctcaaatagtggattgcggtagtaaagattgtgcctgtcttttaaccacgtcaggctcggtggttctcgtgtacccctacagcgagaaatcggataaactctattcacccctacagagagtaaaaagagaatcgccgatgaacaactcatggtggcaggagttaatgcgttttttcctgcaaggaatgacacttaaacagttgattcatatgctaatcatcctgatcgtattgattattgttatgccggtaagcgtaaaagaatggataaacctgcataatccagaaatacttcctcattactggatgtattacatcctgttgttctgcgttagctatgtgcttaacggtgttgttaattccgtttatcacgctgttactgaaagaattgaggcatcaactgctcagcggcgtaaggacagagaagaaaaagtcgttcgggatttgtttgattcgttaactcttggagaaagagcgtatttggcattcgctgtagccgctaataaccagctaaagacagaaaagggaagccctgaagcaatttcattgctcaaaaaagggattatcactcgattgccttctgctattggatatcctgatattgaccgttttattatcccggaaaagtattttaatgagtgctacatgagatttgccgggaagtcagacattcttatgaatgaacttattgtacaggacgaacagctcaaaaaataacgacttaaccgacaaataccttacctcgctgttatttgtttgctcttacgatgaccagccgcgtaaagtgctacgcctggaagaagtacagatcctccttcaacttccttctgacgcgttccggcaagcgaaatggctttggtgacacggtcaattcttttggctttaacttcctgagaagcatcaggagcatcgcagccaaaaattgaatcgatgatattgcagatggtgtcgcgctctatggctagctttctgcgccgctcatgacggcgagttttagcattgcctgcaaacgttgacttcccgtaggtgataaccgtcatgatttaatcctcatgtgaaatggctttggtactggcgccggaacctgtctcaatttccggatttcaagtggcttctcagtccggcccgatcggtacagctagaggcctaagctccaccacacgccagtccaaaccaatctcgtttggtatttgttcgcgctttgtcagcgcatcatcgaagttaaagagcgttgcctttccgtttggctaccagcgtcctgctgatggctaaaatttaagacttcttaattaaatggtcaagtgtatttttgaagaaaacttaaatattttatcgttacttaagtttttatttgatttttaaaggaaaatgtagtgtgaggggcgggtgccccttatggaagatttgcgagttttgcgtcaacaactacgccaatgattttgcagtttccgttgatttctatcatcggatattgtgggtttaatggttttaaaaactttcggcctgcatccataactaattttttgaatgtggcctcgttttcaccttctaattttgcaacaaccagcttgccgtttcttggttcgacttcgggatcaaccagaattatcattccttctggaatgcttaaccctgccggtgctgtcatagagtcaccttggacatcaagccaaaatgaatcttctgaacaatctacagtggtgtcgtgccagttctctatcgcgcgcttgtgataaggttctacagcttccatccattgccctgcgcttacccaactgataagagggtatgatcctcttggctcatgcctactatgataggcaacgtttgtctggcttaaatctcctttcagcaaatagtcaggggagcactgaagagccttcgaaagtgccaacaggttctccccatttggctcagtctccgagcgctcccattgcgatattgcaacattagacactcccaccatcttaccaagagcggcttgtctaatcttgagtttttttcttcgagcgcgaatacgctcacccatcaattgtgtattcatagttaagtcatcttaaataaacttgactaaagattcctttagtagataatttaagtgttctttaatttcggagcgagtctatgtacaagaaagatgttatcgaccacttcggaacccagcgtgcagtagctaaggctttaggcattagcgatgcagcggtctctcagtggaaggaagttatcccagagaaagacgcataccgattagagatcgttacagctggcgccctgaagtaccaagaaaacgcttatcgccaagcggcgtaagcaaaacgctctttaccaatctgaaccgccgacaacgcggtaaacctatttcaaagcgcatcaacgaatgcgcacaactaactattaactacaggaatgttcacatatggaactcacaagcactcgcaagaaagccaacgcaattaccagcagcatccttaaccggatagctattcgtggacagcgtaaagtcgctgatgcgttaggcattaacgaatctcaaatttcacgatggaaaggcgatttcattccgaagatggggatgttattggcggttctggagtggggtgtcgaggatgaggagttggcagaactggcaaagaaagttgcgcatctgctgacaaaagaaaagcctcaagactgcgggaacagttttgaggcctgatgtagaaagactggatcaatccacaggagtaattatgccaaaacaactcagtcctgaccaggacaaattacacaaaaacatactacgtgatcggttcttatccagcttcaaacagcctggtcgatttcgggctgagttggagaaagtgaagctaatactgaagaggaaaggtcatgagtaatcttgcaacagttacaccgataaaacctcatctggaggttgtggagcatcgcgtggcagaactcgacgatggctacacccggactgcaaatacactgctggaagctgtcatgctttctgggcttactcaacatcagctactgattgttatggctgtgtggcgcaagacatacggttataacaaaaaaatagattggatcggaaatgaacagttcgctgaactcactggcatggcgccaaccaaatgttctaccgccaaaaacgagcttatcagaatgggggttctcactcaggtggggcgtcaggttggtatgaataaaaatatttccgagtggaagacgaaggttaacggattcggtaaaacatttaccagatcggtaaaactaaccttcaccaaatcggtaaaaaccaatttaccgaatcagtcaaacacaaaagacaatatacaaaagacaataaatacaaatacccccttaccccctaaagggggatgcgatgaaggttctaaacctgaaaagcgaaaacctaccaagattaactacagcgaatatcttgctgcctacaacgagattgttggtgacagactcccacatgcagtggaggtcaattctgaacgacaacgcaagttgaaaaagctgattgattcactggcaaccaaaaacatcgacggattccgggcatacgtcaaagcgttcatggcagcagccagaccattccatttcggtgataacgaccgtgactgggtagctaattttgattatctgctacgcccgaaagtactgatagcaattcgtgagggaacactatgagacaggatatcgaggcgagcgttatcggtggcttgctgattggcggattaacaccaaccgccagtgacgttctggcaacactggagcctgaagcattctcaattccgctctaccggaaagcttttgaagttattcgaaagcaggccagaaacaggaacctgattgatggactgatggtggccgaggagtgcggggatgaatacgcaacggcggtgatgatgactgcgcggtcatgtcccagcgctgcaaacctgaaaggttatgccggaatggttgcagacagttatcaacggcgtcaggttttacagctactggatgagatgcgggagccaatcagtaacggcacgctggacgcatcaggcagagcgatggacgagcttgtaaagcgcctgtcatccatcaggaagccgcggaacgaggttaaacctgtgcgactgggtgaaatcatcaatgactacactgacacgcttgacaggcgtctgaggaacggagaagagtcggataccctgaagaccggaatcgaagagcttgacgctatcaccggagggatgaacgcagaagaccttgtgattattgctgctcgtccaggtatgggtaaaaccgaactggcgctgaagatagccgaaggcgtggcaagtcgtgttattcctggttctggcgtccggcgcggtgtgttgattttctcgatggaaatgagcgccattcaggttgttgagagagggattgccggcgcaggaatgatgtcggtcagtgtgctgcgtaacccgtcacgtatggacgatgaaggatgggcgagagttgcaagcgggatgaagttgctggcagagctggatgtgtgggtagttgacgcatcgcgtttgtctgtcgaagaaatcaggtccatttccgaacgccacaagcaggagcatcctaatctgtcactgattatggctgactatctcgggctaattgagaaaccaaaagcggaacgtaatgacctcgccatagcacatatctccggtagcctgaaagcgatggcgaaagacctgaaaactccagttatctccctaagccagctctcccgcgatgttgagaagcggccaaacaagcgcccgacaaacgcagatttgcgggattcaggaagcattgaacaggacgcagactcaatcatcatgctctatcgggaagcggtatatgacgagaacagtagcgccgcgccatttgctgaaatcatcgtgacgaaaaaccgttttggctcgcttggtacggtttaccagcggttctgcaacggacactttgttgcatgtgaccaggacgaagccagacagatttgcacggcatcaaatgcacctgctggacgcagaaagcgatatgcacaaggggctgacgtatgactatttacatcactgagttggtaacaggcctgctggtaatcgcaggcctttttatttgggggagagggaagtcatgaaaaaactaacctttgaaattcgatctccagcacatcagcaaaacgctattcacgcagtacagcaaatccttccagacccaaccaaaccaatcgtagtaaccattcaggaacgcaaccgcagcttagaccaaaacaggaagctatgggcctgcttaggtgacgtctctcgtcaggttgaatggcatggtcgctggctggatgcagaaagctggaagtgtgtgtttaccgcagcattaaagcagcaggatgttgttcctaaccttgccgggaatggctttgtggtaataggccagtcaaccagcaggatgcgtgtaggcgaatttgcggagctattagagcttatacaggcattcggtacagagcgtggcgttaagtggtcagacgaagcgagactggctctggagtggaaagcgagatggggagacagggctgcatga |
