# Supplementary figures and images for: Stable Neutralization of a Virulence Factor in Bacteria Using Temperate Phage in the Mammalian Gut
Source: mSystems. 2020 Jan 28;5(1):e00013-20. doi: 10.1128/mSystems.00013-20 (PMC6989128; doi:10.1128/mSystems.00013-20)

non-lysogen

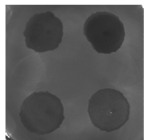

$\lambda$  lysogen

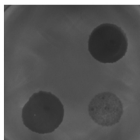

$\lambda$ BH1 lysogen

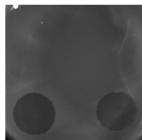

$\lambda$ BH2 lysogen

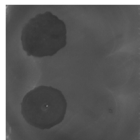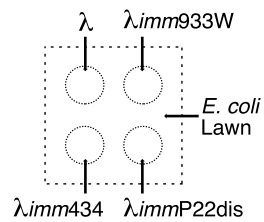

Supplement: FIG S4 [file mSystems.00013-20-sf004.pdf]

### Nonlysogen

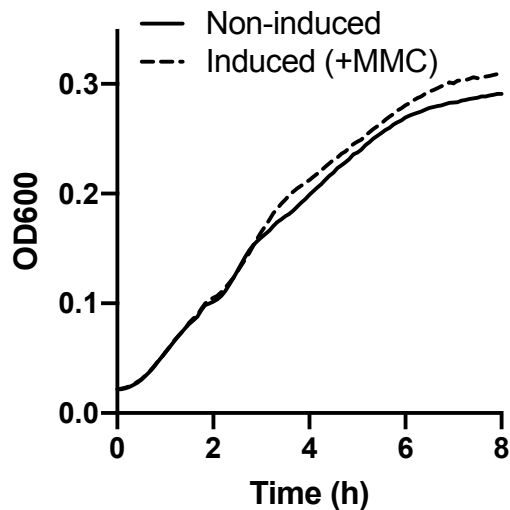

### 933W lysogen

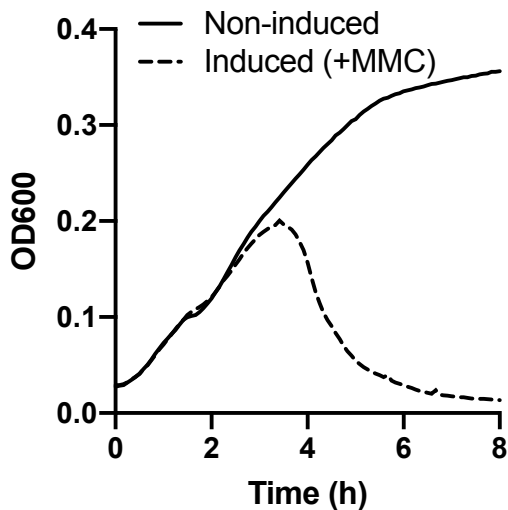

### $\lambda$ BH2 lysogen

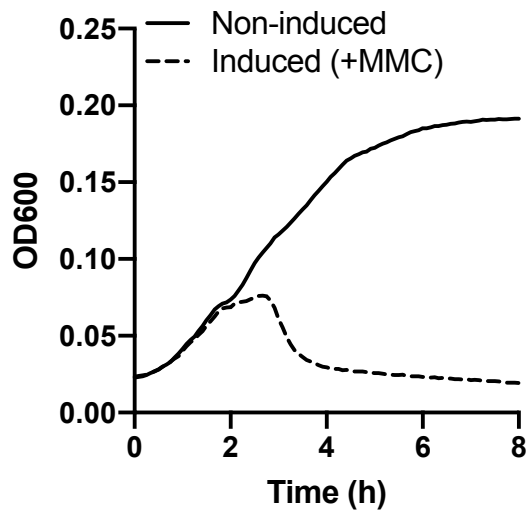

### $\lambda$ BH2 / 933W-polylysogen

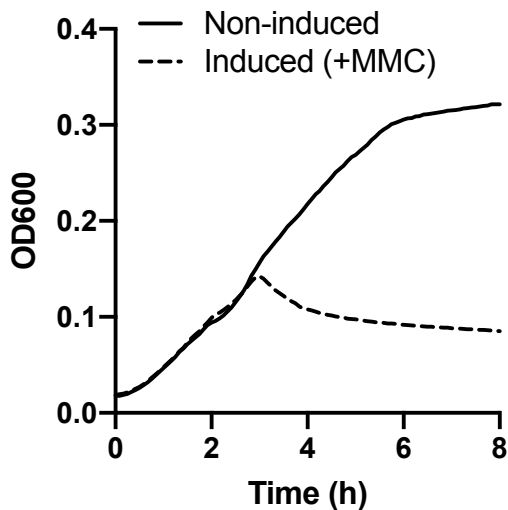

Supplement: FIG S5 [file mSystems.00013-20-sf005.pdf]
