# Supplementary material for: Stable Neutralization of a Virulence Factor in Bacteria Using Temperate Phage in the Mammalian Gut
Source: mSystems. 2020 Jan 28;5(1):e00013-20. doi: 10.1128/mSystems.00013-20 (PMC6989128; doi:10.1128/mSystems.00013-20)
Supplement: TABLE S1 [file mSystems.00013-20-st001.docx]

| Bacteria | Description | Source |
| --- | --- | --- |
| *E. coli* | Strain MG1655 with spontaneous streptomycin resistance | ATCC |
| *E. coli* C600 | Strain C600 | ATCC |
| *E. coli* ^933W^ | *E. coli* lysogenized by 933W phage derived from E. coli O157:H7 | This paper |
| *E. coli* O157:H7 | Strain edl933 | ATCC |
|  |  |  |
| λ*imm*933W lysogen | *E. coli* lysogenized by λ*imm*933W | This paper |
| λ lysogen | *E. coli* lysogenized by λ | This paper |
