# Supplementary material for: Stable Neutralization of a Virulence Factor in Bacteria Using Temperate Phage in the Mammalian Gut
Source: mSystems. 2020 Jan 28;5(1):e00013-20. doi: 10.1128/mSystems.00013-20 (PMC6989128; doi:10.1128/mSystems.00013-20)
Supplement: TABLE S2 [file mSystems.00013-20-st002.docx]

| Phage | Description | Source |
| --- | --- | --- |
| λ | Wild-type | ATCC |
| λ*imm*933W |  | Gerald Koudelka |
| λ*imm*21 |  | Donald Court |
| λ*imm*434 |  | Donald Court |
| λ*imm*P22dis |  | Donald Court |
| λBH1 | λ; ea47-*ea31::Tn5-933W⋅cI^ind-^* | This paper |
| λBH2 | λ; *ea47*-*ea31::Tn5-933W⋅cI^ind-^; λ⋅sieB-λ⋅ren::P22⋅sieB- P22⋅12* | This paper |

|  |  |
| --- | --- |
